# Probing the spatiotemporal patterns of HBV multiplication reveals novel features of its subcellular processes

**DOI:** 10.1371/journal.ppat.1009838

**Published:** 2021-08-09

**Authors:** Lei Yue, Chang Li, Mingzhu Xu, Min Wu, Jiahui Ding, Jiangxia Liu, Xiaonan Zhang, Zhenghong Yuan

**Affiliations:** 1 Key Laboratory of Medical Molecular Virology (MOE/NHC/CAMS), School of Basic Medical Sciences, Shanghai Medical College, Fudan University, Shanghai, China; 2 Research Unit, Shanghai Public Health Clinical Center, Fudan University, Shanghai, China; 3 Centre for Research in Therapeutic Solutions, Biomedical Sciences, Faculty of Science and Technology, University of Canberra, ACT, Australia; The Pennsylvania State University College of Medicine, UNITED STATES

## Abstract

Through evolution, Hepatitis B Virus (HBV) developed highly intricate mechanisms exploiting host resources for its multiplication within a constrained genetic coding capacity. Yet a clear picture of viral hitchhiking of cellular processes with spatial resolution is still largely unsolved. Here, by leveraging bDNA-based fluorescence in situ hybridization (FISH) combined with immunofluorescence, we developed a microscopic approach for multiplex detection of viral nucleic acids and proteins, which enabled us to probe some of the key aspects of HBV life cycle. We confirmed the slow kinetics and revealed the high variability of viral replication at single-cell level. We directly visualized HBV minichromosome in contact with acetylated histone 3 and RNA polymerase II and observed HBV-induced degradation of Smc5/6 complex only in primary hepatocytes. We quantified the frequency of HBV pregenomic RNAs occupied by translating ribosome or capsids. Statistics at molecular level suggested a rapid translation phase followed by a slow encapsidation and maturation phase. Finally, the roles of microtubules (MTs) on nucleocapsid assembly and virion morphogenesis were analyzed. Disruption of MTs resulted in the perinuclear retention of nucleocapsid. Meanwhile, large multivesicular body (MVB) formation was significantly disturbed as evidenced by the increase in number and decrease in volume of CD63^+^ vesicles, thus inhibiting mature virion secretion. In conclusion, these data provided spatially resolved molecular snapshots in the context of specific subcellular activities. The heterogeneity observed at single-cell level afforded valuable molecular insights which are otherwise unavailable from bulk measurements.

## Author summary

HBV is a hepatotropic, enveloped virus with a partially double-stranded relaxed circular DNA genome. Studies on the molecular biology of HBV mainly rely on biochemical extraction and bulk quantification methods. Detailed spatiotemporal information on virus components in subcellular context is still lacking. Here, we re-evaluated the reproduction schemes of HBV by fluorescence in situ hybridization (FISH). We visualized cccDNA minichromosome formation in an epigenetic context, identified pgRNA associated with actively translating ribosomes and capsids. Moreover, the active participation of microtubules in nucleocapsid transport and MVB-mediated virion secretion was identified. These observations have broad implications for understanding the HBV replication cycle and may facilitate the identification of novel antiviral targets.

## Introduction

HBV is a hepatotropic, enveloped virus of the hepadnaviridae family with a partially double-stranded relaxed circular DNA (rcDNA) genome [1]. The rcDNA in incoming virion is repaired by cellular enzymes and transformed into covalently closed circular DNA (cccDNA), the template for all HBV transcripts, including pregenomic RNA (pgRNA) and subgenomic RNAs [2]. The pgRNA serves not only as the template for viral reverse transcription but also as the messenger encoding HBcAg (Core) and the polymerase (Pol) essential for its genome replication [3]. Nucleocapsid assembly is initiated by the binding of viral polymerase to pgRNA together with cellular factors such as Heat shock protein 90 (Hsp90) [4]. Subsequently, viral DNA synthesis is initiated, and mature nucleocapsid is then enveloped by viral surface antigens. Cellular microtubules (MTs) network mediates the delivery of nucleocapsid into the nucleus following viral entry [5,6], they were also proposed to be required for nucleocapsid formation [7].

With the aid of a series of classical analytical methods such as ultrafiltration, Southern and Northern blot etc. [1], the aforementioned framework of HBV life cycle was established. However, this picture still lacks many key spatial and molecular contexts which involves essential co-opted host factor, and there is also a general lack of observations at single-cell level in the context of cellular architectures. Previously, by optimizing the viewRNA in situ hybridization assay, we developed an assay for visualizing HBV RNA, DNA and cccDNA in liver specimens and in cell model [8–10]. Here, by combining this assay with immuno-detection of proteins, we were able to analyze the formation of intranuclear minichromosome, viral transcription, translation, packaging and viral egress in a multiplexed and spatially resolved manner. We observed the distinct localization of nuclear HBV DNA within the milieu of K27 acetylated Histone H3 and RNA Polymerase II (Pol II). In addition, we surveyed the occupancy of ribosome and capsids on pgRNA and confirmed the mutually exclusive nature of pgRNA translation and encapsidation. In addition, we inferred from the quantitative data the relatively quick pgRNA translation and slow capsid maturation process. Finally, we found that although nucleocapsids can still be assembled after MTs disruption, their transport was arrested in the peri-nuclear region. Moreover, virion morphogenesis and release via the MVB route were significantly impaired as vesicle fusion was disrupted in the absence of functional MTs. The ability to visualize HBV nucleic acids within sophisticated subcellular architectures enabled us to observe cell-to-cell variability of HBV infection and to probe the subcellular as well as molecular details of viral activity.

## Results

### Specific and sensitive FISH imaging of HBV RNA and DNA

We first used the QuantiGene ViewRNA ISH detection system to specifically detect HBV DNA and RNA in HepAD38 (DOX-) cells, in which robust HBV replication can be switched on by removal of doxycycline [11]. The design of the probe sets was similar to our previous report [8]. We designed probe set 1 for visualizing pgRNA/ plus-strand DNA (+DNA) and probe set 2 visualizing for minus-strand DNA [(-) DNA] (S1 Fig). It should be noted that although preC mRNA can also be detected by probe set 1, quantitative analysis indicated that the contribution of preC mRNA was minute in HepAD38 and HepG2-NTCP infection system (S2A Fig).

**Fig 1:**
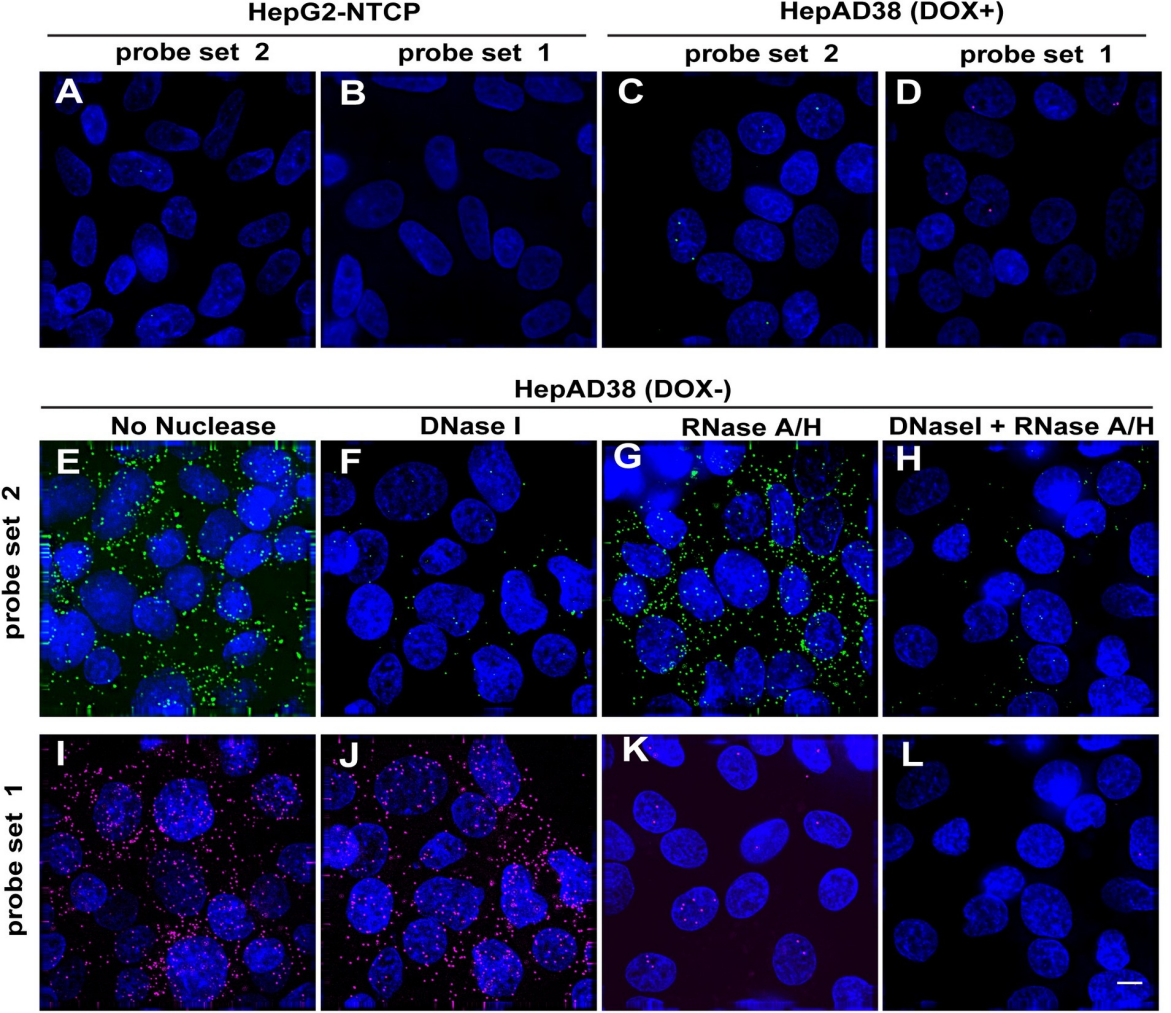
HBV RNA and DNA detection with specific probe in HepAD38 (DOX) cells. HepG2-NTCP (A-B) and HepAD38 (DOX-) cells (E-L) were maintained in the doxycycline-free medium, HepAD38 (DOX+) cells (C-D) were maintained in the doxycycline medium for 7 days, then cells were fixed and stained. Prior to probe labeling, cells were treated with buffer alone (A-D, E, I), DNase I (F, J), RNase A/H (G, K), or DNase I+ RNase A/H (H, L). Probe set 2 was used for HBV (-) DNA (green); Probe set 1 was used for HBV (+) DNA and pgRNA (purple). These two probe sets were mixed and hybridized with cells and signals were captured in two independent channels (Probe set 2 Cy5, Probe set 1 Cy3), representative images were shown. Scale bar, 4 μm.

Using this assay, virus-specific signals were detected in HepAD38 (DOX-) cells (Fig 1E and 1I) whereas virtually no signal was observed in HepG2-NTCP cells (Fig 1A and 1B). Essentially no pgRNA signals were detected in HepAD38 (DOX+) cells, due to the shutdown of pgRNA transcription (Fig 1D). A few nuclear puncta ranging from 1 to 3 per cell were observed in HepAD38 (DOX+) cells which is due to the integrated HBV DNA (Fig 1C and 1D) in accordance with a previous report [10]. The molecular specificity of the signals was confirmed by pre-treatment of nucleases. HBV (-) DNA signals were rarely detected by probe set 2 in cells with DNase I treatment (Fig 1F), the remaining puncta might be caused by the limited activity of DNase I and capsid protection. By contrast, RNaseA/H did not affect the signal (Fig 1G). The combined digestion by DNase and RNases generated results that were similar to DNase I treatment alone (Fig 1H). In comparison, abundant FISH signals were detected by probe set 1 with DNase I treatment indicating that the majority of the signals were derived from pgRNA (Fig 1J). RNase A/H digestion eliminated most of the signals except some discernible spots within the nuclei suggestive of intranuclear DNA (Fig 1K). Indeed, DNase and RNases co-treatment further erased nuclear signal (Fig 1L). Hence, the ViewRNA FISH detection system allowed us to study the intracellular distribution of HBV DNA and RNA with molecular specificity.

### Kinetics of HBV DNA and RNA accumulation during infection

**Fig 2:**
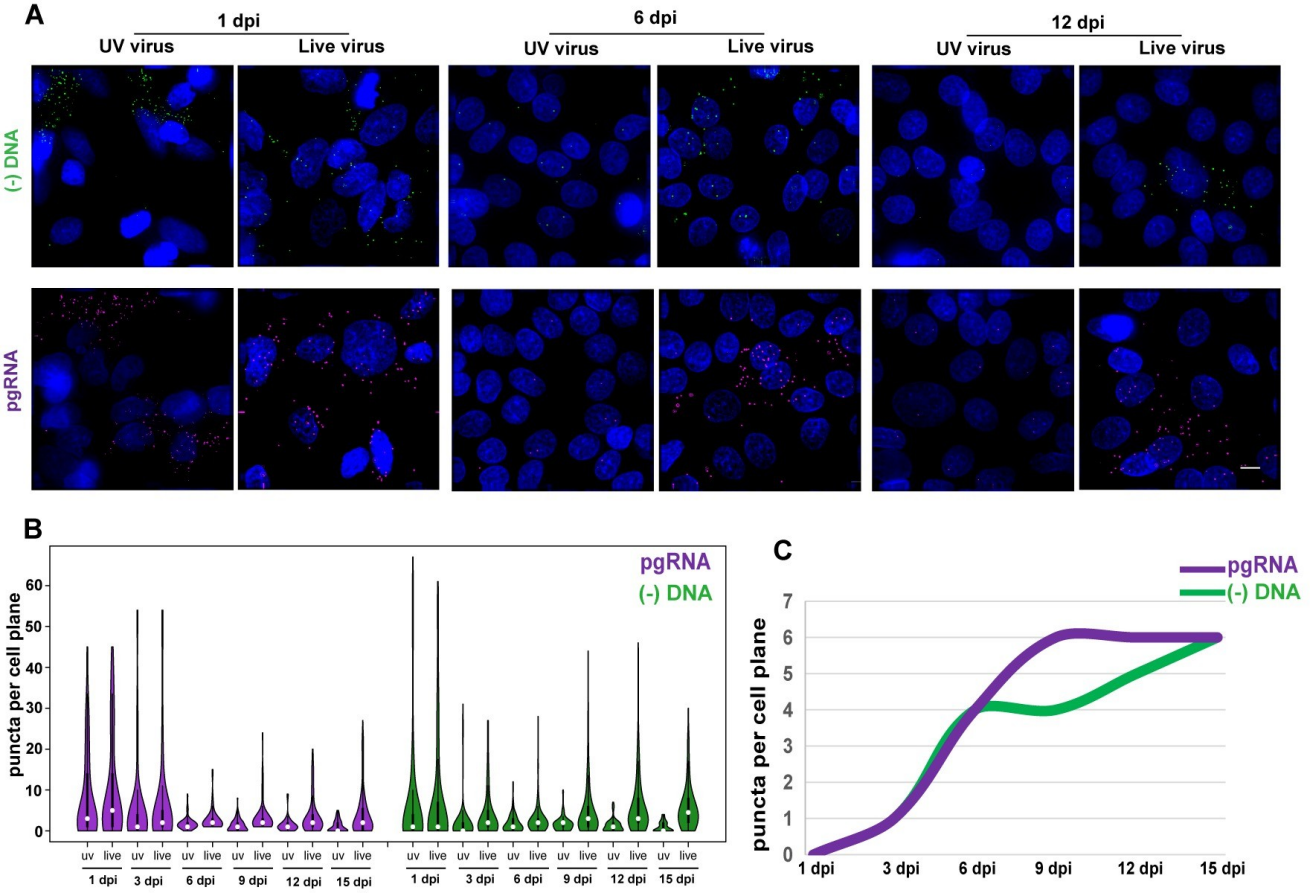
Kinetics of HBV DNA and RNA accumulation during infection. (A) HepG2-NTCP cells were infected with UV-irradiated or untreated HBV at MOI = 1000. At the indicated times post-infection, cells were fixed and processed for FISH detection. Scale bar, 4 μm. (B) Individual pgRNA and (-) DNA puncta for each time point were quantified and graphed using the R package. More than 100 cells per group were counted. (C) The median of the true pgRNA and (-) DNA signal (live virus signal subtracted by UV virus signal) over the indicated time post-infection were plotted using the smooth graph function in Microsoft Excel.

We next examined the kinetics of HBV (-) DNA and pgRNA in HBV infected HepG2-NTCP cells. Over a time-course after virus inoculation, we visualized and quantified HBV (-) DNA and pgRNA at the single-cell level (Figs 2A and 2B and S2B). We used UV-irradiated virus as a control to evaluate how much FISH signal was derived from inoculum at each time point. The (-) DNA and pgRNA puncta were comparable between live virus infection and UV-irradiated virus control at 1 dpi and 3 dpi, which were consistent with a previous report [12]. From 6 dpi to 15 dpi, the (-) DNA and pgRNA signals were rarely detected in UV-irradiated virus group (Fig 2A and 2B) suggesting that the signals obtained from this time window were from active replication.

Next, we quantified the (-) DNA and pgRNA puncta per cell on day 6, 9, 12 and 15 post infection (S3A and S3B Fig). It was found that the number of (-) DNA and pgRNA molecules per cell was highly variable. A majority of them had less than 5 puncta per cell and very few had abundant dots. Obviously, they were not normally distributed. By parametric distribution fitting using fitdistrplus, these data were found to be best fit to the geometric distribution (S3A and S3B Fig, boxed region in the upper-right). Interestingly, this distribution feature remained unchanged from day 6 to day 15. We then used the median of the true signal (live virus signal subtracted by UV virus signal) at different time points to generate the kinetic curve of the viral nucleic acid (Fig 2C). We observed that HBV pgRNA signal steadily increased from day 1 to 9 dpi (median, 6 puncta per cell) and remained steady thereafter. In comparison, HBV (-) DNA signal experienced a lag from 6 dpi to 9 dpi (median, 4 puncta per cell) and then continued to increase slowly from 9 to 15 dpi (median, 6 puncta per cell).

We also used bulk measurement to validate our FISH data. We detected HBV nucleocapsid DNA, and RNA with Southern and Northern blot. In accordance with a previous report [12], significant amount of inoculated DNA was detected on day 1 and 3 post infection (S2C Fig). Overall, the kinetics obtained from bulk measurements (S2C and S2D Fig) correlated well with the proliferation trend of DNA and pgRNA signals detected by FISH.

We next sought to determine whether this same technique could be used to evaluate the effects of antivirals. We infected HepG2-NTCP cells with HBV for 6 h, and at 3 dpi, cells were treated with Interferon-α, a pleotropic antiviral cytokine, or Entecavir, a nucleoside analog, and fixed at 6, 9, and 12 dpi for FISH analysis (S4A Fig). Interferon-α had a significant inhibitory effect on both (-) DNA and pgRNA signals (S4B and S4D Fig), it also reduced the proportion of viral nucleic acid positive cells (S4C and S4E Fig). By contrast, Entecavir only inhibited (-) DNA (S4B and S4C Fig) but not pgRNA signal (S4D and S4E Fig). These data were consistent with their mode-of-actions [13].

### Visualizing the key nuclear molecules crucial for cccDNA assembly and viral transcription

**Fig 3:**
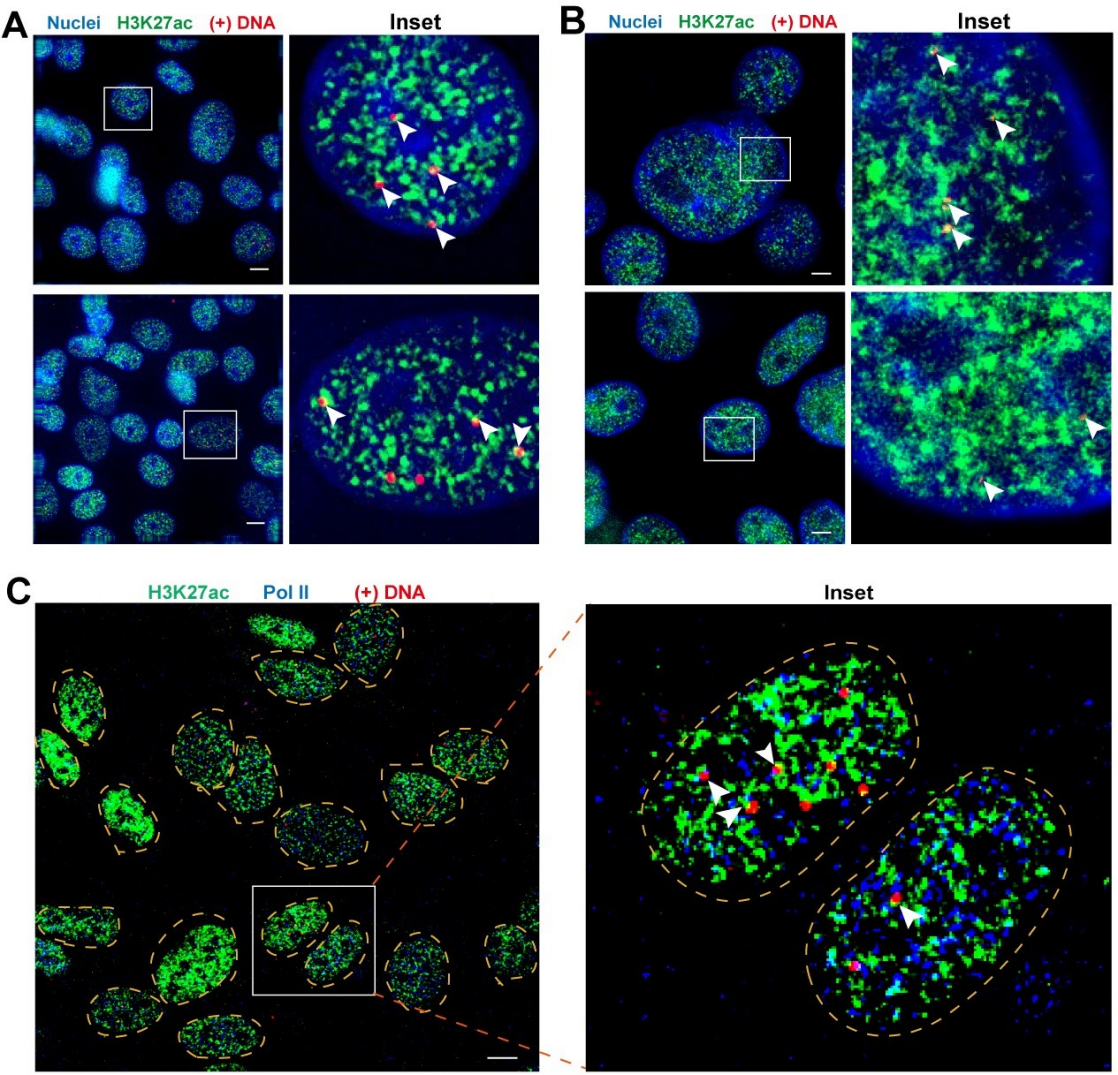
Colocalization of HBV (+) DNA with H3K27ac and Pol II proteins. HepG2-NTCP cells were infected with HBV at MOI = 1000 and at 12 days post-infection the cells were fixed and processed for viral (+) DNA detection followed by immunofluorescence staining for H3K27ac proteins with Alexa Fluor 488 labelled secondary goat anti-rabbit antibody and/or Pol II proteins with CY3 labelled secondary goat anti-mouse antibody. (A)/(C) Wide-field images. (B) Stimulated Emission Depletion (STED) super resolution images. Nuclei borders were represented by yellow dashed lines in (C). Scale bar, 4 μm. Solid white arrows point to (+) DNA colocalized with H3K27ac or with H3K27ac and Pol II proteins.

HBV cccDNA is assembled with histones and other epigenetic factors to form minichromosome as the template for HBV transcription [14,15]. Epigenetic mechanisms, such as post-translational modifications (PTMs) of histone proteins, are crucial in mediating its transcriptional activity [16]. By using probe set 3 (S1 Fig) targeting the gap region of the HBV virus particle genome in conjunction with RNaseA/H treatment, we detected intranuclear (+) DNA in the context of H3K27ac in HepG2-NTCP infection system (Fig 3A) using wide-field microscope. STimulated Emission Depletion (STED) microscopy [17] obtained higher resolution images, which detected smaller irregular-shaped puncta (Fig 3B). Staining of H3K27ac in HBV infected HepG2-NTCP cells were distributed in distinct nuclear regions. Closer inspection of the enlarged areas revealed that the majority of the HBV DNA molecules were surrounded by H3K27ac highly suggestive of chromatinized cccDNA (Fig 3A and 3B, inset white arrows).

To further probe the epigenetic status of these cccDNA, we further detected (+) DNA with H3K27ac and Pol II (Fig 3C), a marker for active transcription. We found that the majority of (+) DNA was in close contact with the “cloud” of H3K27ac and Pol II (Fig 3C, inset white arrows). We used Huygens “Object analyzer” to quantify the spatial proximity between discrete HBV DNA dots and H3K27ac/Pol II foci. The Venn diagram further reflected the geometric relationship among these molecules (S5 Fig). First, 50.3% of the H3K27ac foci and 77% of the Pol II foci co-localized with each other, corroborating the association of histone 3 acetylation and polymerase II initiation and elongation [18]. It is suggested that these co-localized regions may have transcriptional activity [18]. Furthermore, 43% of the cccDNA closely associated with both H3K27ac and Pol II and 46.1% of them colocalized with either of these two markers (with 29.2% H3K27ac or 16.9% Pol II) (S5 Fig). These data suggests that the intranuclear cccDNA are mostly engaged in an epigenetic milieu that is enriched in H3K27ac and Pol II such that a fully chromatinized transcriptional active state can be ensured.

HBx mediated cccDNA de-silencing has been considered the key event for viral transcription, and involves the degradation of Smc5/6 (maintaining the 5/6 complex of chromosomal structure) by recruiting DDB1 associated E3 ligases [19]. We attempted to validate this phenomenon by visualizing HBV (-) DNA and Smc5/6 in complex in primary human hepatocytes (PHH) and in HepG2-NTCP cells (S6B Fig). We observed robust HBV replication as the (-) DNA signal in PHH (median, 23.5 puncta per cell) was significantly higher than that in HepG2-NTCP cells (median, 5 puncta per cell) at 12 dpi (S6C Fig). The subcellular localization of Smc5/6 complex was confirmed by separation of nuclear and cytoplasmic fraction followed by immunoblotting (S6A Fig). In accordance with previous studies [19–21], primary human hepatocytes infected with HBV displayed a loss of Smc6 (S6B Fig, lower panel) whereas uninfected cells displayed discrete nuclear Smc6 puncta (S6B Fig, bottom right, solid white arrow). However, HBV infected HepG2-NTCP cells showed no obvious change in Smc5/6 level (S6B Fig, upper panel, inset, compare DNA positive and negative cells). These data confirmed the effect of HBx on Smc5/6 complex in primary culture. The different behavior of HepG2-NTCP cells in response to HBV infection suggested that the abundance and the regulation of Smc5/6 complex in hepatoma cell lines had been altered. Hence these systems might not be appropriate for studying HBx-mediated transcription activation.

### Pregenomic RNA translation and encapsidation

The pgRNA plays a dual role in HBV replication, not only as the template for core/pol translation, but also for viral genome synthesis. However, the two processes of translation and encapsidation are inherently competitive, given that they share a common precursor [22]. We applied a ribosome-bound nascent chain puromycylation assay to detect pgRNA occupied by translating ribosomes. In the absence of puromycin, no fluorescent signal was detected, while diffuse cytoplasmic signal (green) was readily detected in the presence of puromycin (S7 Fig).

**Fig 4:**
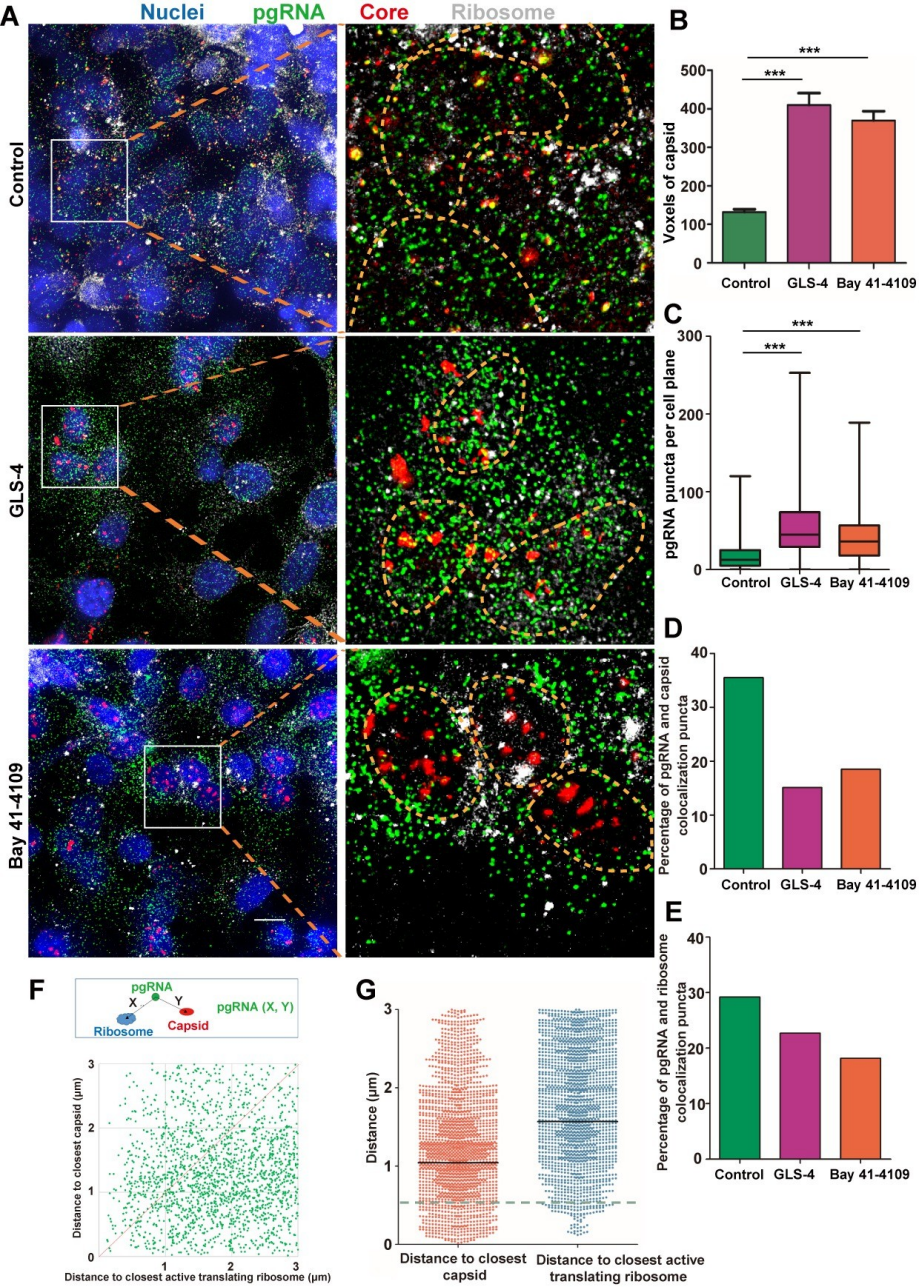
Colocalization of HBV pgRNA with active translating ribosomes or capsid in HepAD38 (DOX) cell. (A) HepAD38 (DOX-) cells were untreated or pretreated with 10 μM GLS4/Bay 41–4109 followed by puromycin (Puro) labeling. Cells were fixed and processed for pgRNA detection followed by immunofluorescence staining for puromycylated ribosomes with Alexa Fluor 488 labelled secondary goat anti-mouse antibody and capsid with Cy3 labelled secondary goat anti-rabbit antibody. Nuclei borders were represented by yellow dashed lines in the enlarged areas. Scale bar, 4 μm. (B) Voxels of capsid were quantified using Huygens. (C) Puncta of pgRNA were quantified using FISH-quant. More than 200 cells per group were counted. (D, E) Percentage of pgRNA colocalized with capsid or puromycylated ribosomes were quantified by Huygens. (F) Distance between pgRNA and capsid (Y) or puromycylated ribosomes (X) were shown as a two-dimensional plot. (G) The distribution of aforementioned X and Y values were shown as a scatter plot with marked mediums. A dashed line of 0.5 μm, the threshold for defining colocalization, was shown. ***P < 0.001 (Mann-Whitney U-test).

To validate the feasibility of evaluating pgRNA encapsidation using fluorescent microscopy, we tested whether CpAMs (capsid allosteric modulators) could disrupt the spatial proximity between capsid and pgRNA. CpAMs are able to induce aberrant capsid that are unable to encapsidate pgRNA [23]. We used Bay 41–4109 and GLS-4 [24,25], members of the class I CpAMs which misdirect core protein to form polymer aggregates and subsequently degraded. As expected, HepAD38 cells treated with either Bay 41–4109 or GLS-4 resulted in 0.81 log_10_ IU/mL and 0.6 log_10_ IU/mL reduction of extracellular HBV DNA (S8A Fig), 2.13 log_10_ IU/mL and 1.64 log_10_ IU/mL reduction of encapsidated-pgRNA (S8B Fig) and 1.09 log_10_ IU/mL and 1.32 log_10_ IU/mL reduction of encapsidated-DNA (S8C Fig). Almost no detectable core particle DNA was detected in the presence of CpAMs by Southern blot (S8D Fig). Puromycylation assay in combination with pgRNA FISH and core immunofluorescence in HepAD38 cells (Fig 4A) revealed 3 and 4 fold increase in the volume of capsid puncta when cells were treated with the two CpAMs (Fig 4B). In addition, the number of pgRNA puncta (median value) increased by 3.6 and 2.9 fold (Fig 4C), and the number of pgRNA puncta co-localized with capsid reduced by 57.3% and 47.9% (Fig 4D). Meanwhile, the number of pgRNA puncta co-localized with actively translating ribosome reduced by 22.4% and 37.8% (Fig 4E). These results confirmed that CpAMs significantly disrupted the encapsidation of pgRNA, resulting in large capsid aggregates in the cytoplasm. It also verified the reliability of our methodology to study molecular events related to pgRNA processing.

To quantitatively analyze the colocalization between pgRNA and capsid or puromycylated ribosomes, we defined the distance between pgRNA and its closest puromycylated ribosome as X, the distance between pgRNA and its nearest capsid as Y. This resulted in a 2D scatter plot showing that more dots were closer to the X-axis (Fig 4F). This suggests that the distance between pgRNA and capsid was mostly closer than between pgRNA and puromycylated ribosomes (Fig 4G). We further calculated the ratio of pgRNA colocalizing with capsid or ribosome in cytoplasm (Table 1), in which colocalization was defined as less than 500 nm between two objects. Around 3.4% of the pgRNAs colocalized with puromycylated ribosomes whereas around 13.2% of pgRNAs were colocalized with core particles. The ratio of pgRNA for encapsidation to pgRNA for translation was 3.9 to 1. Venn diagrams further reflected the relationship among pgRNA, capsid and translating ribosome (S9 Fig). It was found that 35.9% of core particles were colocalized with pgRNA and 64.1% of them were empty capsid or dimeric and oligomeric core proteins (S9A Fig), while owing to the weak and often diffuse signals generated by the core dimers or oligomers, empty capsid should contribute to the most proportion. Statistics showed very few (0.8%) of pgRNA puncta was colocalized with both capsid and ribosome. We reasoned that this was caused by coincidence since it was close to the random chance of triple positive, i.e., 13.2%*3.4% = 0.45%.

**Table 1:** Distribution ratio of different forms of pgRNA in HepAD38 cell and in HepG2-NTCP infection system.

pgRNA	Capsid	Ribosome	Proportion in HepAD38 cell	Proportion in HepG2-NTCP infection system
+	+	+	0.8%	2.0%
+	+	-	13.2%	34.8%
+	-	+	3.4%	2.7%
+	-	-	82.6%	60.5%

**Fig 5:**
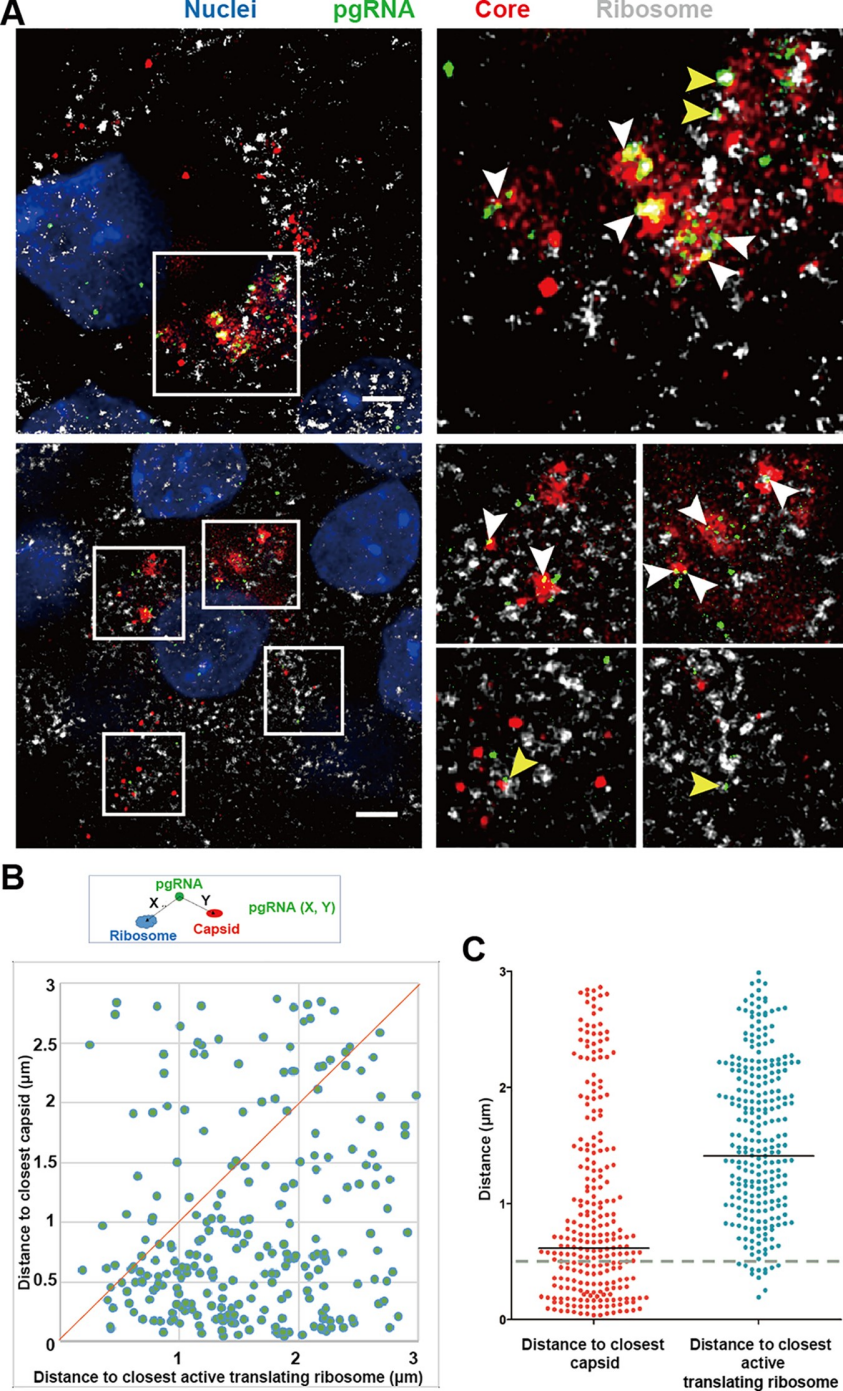
Colocalization of HBV pgRNA with actively translating ribosomes and capsid in the HepG2-NTCP infection system. (A) HepG2-NTCP cells were infected with HBV at MOI = 1000 and at 9 days post-infection the cells were fixed and processed for pgRNA detection followed by puromycin (Puro) labeling for puromycylated ribosomes and capsid. Colocalization of pgRNA and capsid were presented by white arrows. colocalization of pgRNA and actively translating ribosomes were presented by yellow arrows. Scale bar, 4 μm. (B) Distance between pgRNA and closest puromycylated ribosomes (X), between pgRNA and closest capsid (Y) were shown as a two-dimensional plot. (C) The distribution of aforementioned X and Y values were shown as a scatter plot with marked mediums. A dashed line of 0.5 μm, the threshold for defining colocalization, was shown.

Similar experiments were performed in HBV-infected HepG2-NTCP cells. We observed the same trends as in HepAD38 (DOX-) cells, with 34.8% of the pgRNAs puncta colocalized significantly with capsid (Fig 5A, white arrows) and only a few (2.7%) with puromycylated ribosomes (Fig 5A, yellow arrows). The distance between pgRNA and capsid was mostly closer than that between pgRNA and ribosomes (Fig 5B). The number of pgRNAs occupied by capsids (~34.8%) was 12.9 times the number occupied by ribosomes (~2.7%) (Table 1). Furthermore, 90.1% of the capsids were empty whereas 9.1% of them harbored pgRNAs (S9B Fig). Interestingly, the ratio of triple positive (2.0%) was significantly larger than estimate of random coincidence (34.8%*2.7% = 0.9%). Indeed, we observed that a significant portion of pgRNA, capsid and ribosomes were clustered in proximity as observed in Fig 5A.

### Nucleocapsid transport rather than assembly requires microtubules integrity

The assembly of pgRNA-containing capsid (immature nucleocapsid) depends on specific viral and host factors. A previous study proposed that microtubule disassembly could inhibit nucleocapsid formation [7]. To further investigate the role of MTs in capsid assembly, we examined the distribution of viral nuclei acids, HBcAg and α-tubulin in HBV replicating cells with microtubule polymerization inhibitors (MTIs), i.e., Nocodazole and Vinblastine.

**Fig 6:**
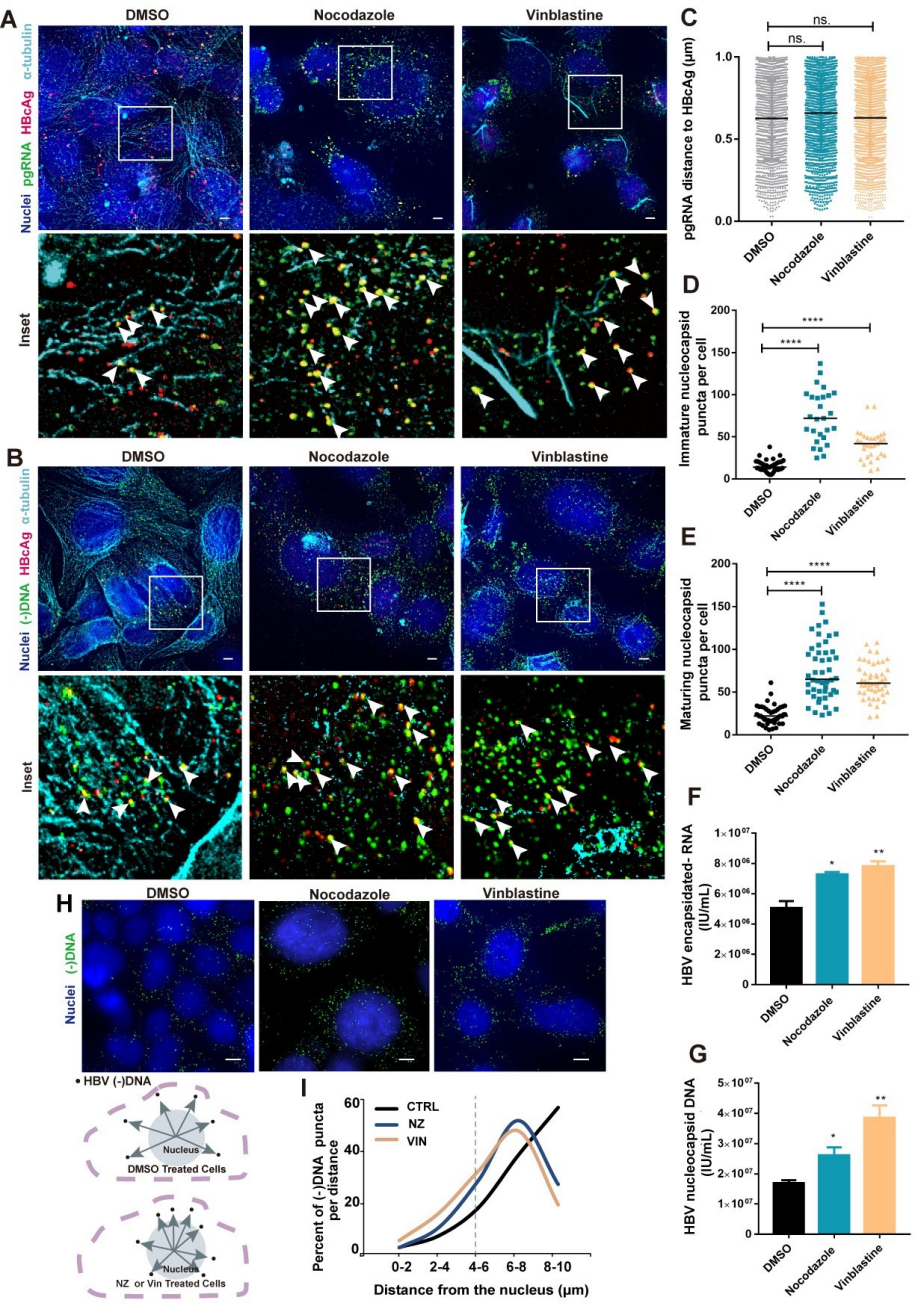
Effects of MTs disruption on HBV nucleocapsid formation. HepAD38 cells were treated with Nocodazole or Vinblastine for 24 h before fixation and processed for HBV pgRNA (A) or (-) DNA (B), HBcAg and α-tubulin detection. White arrows indicate the pgRNA or (-) DNA were colocalized with HBcAg. Scale bar, 4 μm. (C) Analyzing pgRNA distance to its closest HBcAg within 1 μm using Huygens in FISH images. (D, E) Quantitative analysis of HBV immature and maturing nucleocapsid puncta in single cell using Huygens in FISH images. More than 15 cells per group was counted. Intracellular HBV encapsidated pgRNA (F) and nucleocapsid DNA (G) were quantified by real-time PCR. (H, I) Representative images, schematics and measurements of cellular distribution of viral (-) DNA compared to the nucleus in DMSO or MTIs-treated cells using Huygens. More than 15 cells per group was counted. NZ, Nocodazole; VIN, Vinblastine. *P < 0.05, **P < 0.01, ****P < 0.0001. ns: no significance. (C-E): Mann-Whitney U-test; (F, G): Student’s *t*-test, the data are representative of three independent replicates.

Viral immature and maturing nucleocapsids were observed in close proximity to microtubule structure (Fig 6A and 6B, left), a finding consistent with the previous report. MTIs treatment broke down the filamentous structure of MTs into bundles of dense tubules (Fig 6A and 6B, middle and right), but nucleocapsids were still frequently found near them (Fig 6A and 6B, middle and left, white arrows). Moreover, the colocalization between pgRNA and HBcAg was not affected, which was confirmed by molecular statistics (Fig 6C). Therefore, microtubule disruption had minimal effect on nucleocapsid formation itself. Of note, MTIs had negligible effect on cell viability at their working concentrations (S10A and S10B Fig).

We then quantitatively analyzed viral nuclei acids puncta, as well as nucleocapsid puncta at single-cell level. We found significant intracellular accumulation of viral pgRNA and (-) DNA (S10C and S10D Fig) with MTIs treatment, similar trends were observed for nucleocapsids (Fig 6D and 6E). Bulk measurement of intracellular encapsidated pgRNA (Fig 6F) and DNA (Fig 6G) confirmed the imaging data. More importantly, we found that a significant number of nucleocapsids were retained in the perinuclear region under MTIs treatment (Fig 6H). We measured the distance from nuclear center to (-) DNA and plotted its distribution frequency (Fig 6H and 6I) which showed a striking difference after MTIs treatment. In control group, 22% of (-) DNA located in 2–6 μm of the nuclear center, Nocodazole and Vinbalstine treatment increased these figures to 40% and 36% respectively (Fig 6I). Taking these results together, we concluded that MTs plays a significant role in nucleocapsid transport from perinuclear region to cell periphery.

### Microtubule integrity facilitates virion secretion by promoting MVBs biogenesis

The release of complete virions requires the binding of mature nucleocapsids to the preS domain of LHBsAg and was further facilitated by MHBsAg and HBsAg [26–28]. The roles of MTs on virion morphogenesis is largely unknown. It was found that in MTIs-treated cells, intracellular LHBsAg (gp42 and p39) increased by 53% and 59%, respectively (S11A Fig). Further measurement showed that MTIs could significantly reduce extracellular HBsAg but not HBeAg (S11B Fig). Similarly, the extracellular HBV DNA was also reduced (S11C Fig). By in situ detection of viral (-) DNA and preS1, only a few double-positive virion signals (median, 4 puncta per cell) were seen in most cells (S11D Fig, upper panel). However, we observed a significant increase (Nocodazole 2.2 fold, Vinblastine 3 fold) in the number of intracellular virion puncta (S11D, middle and bottom, and S11E Fig) when treated with MTIs. These data suggest that microtubule integrity was crucial for assembly and release of mature virions.

**Fig 7:**
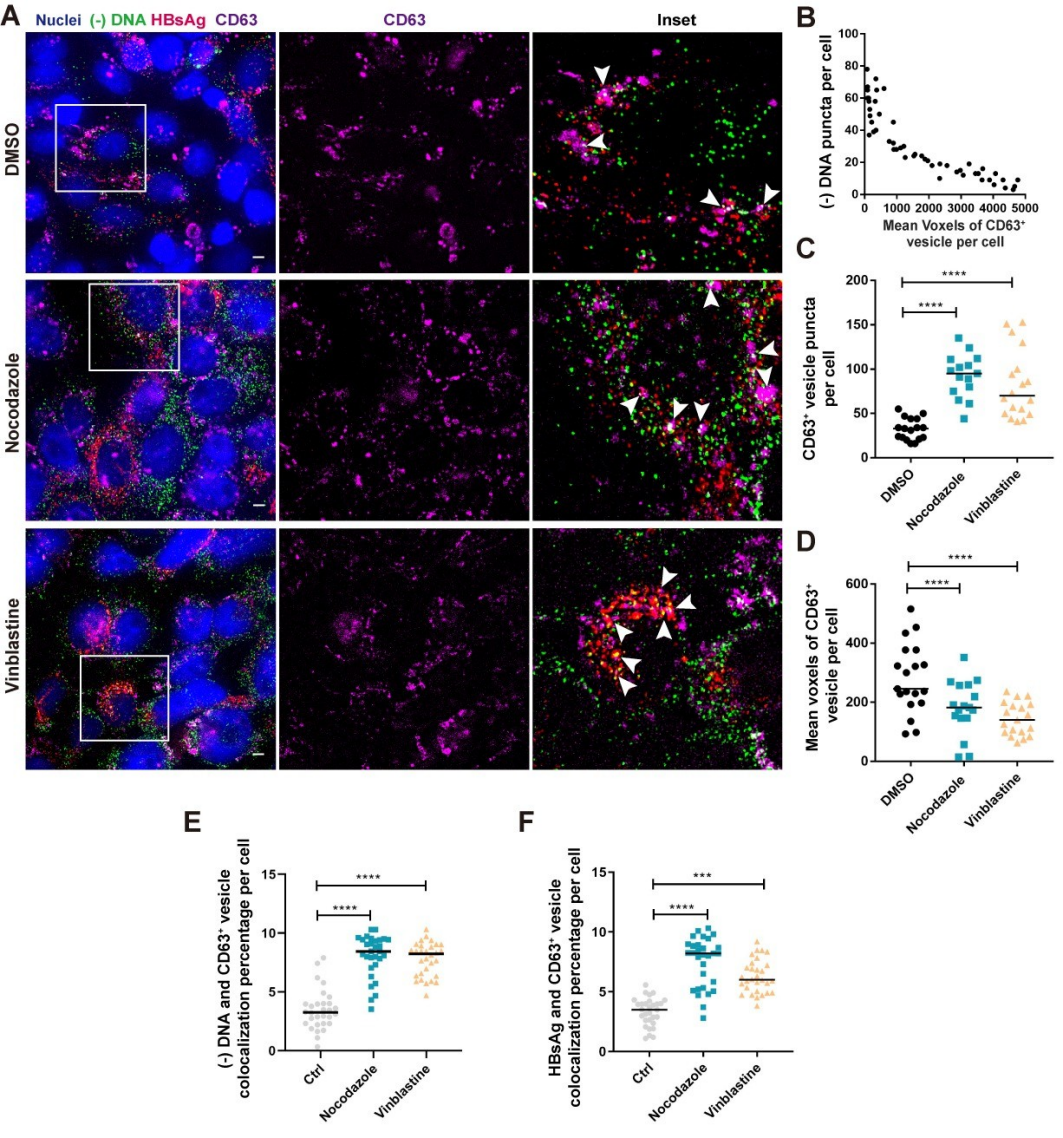
MTs orchestrate HBV virion secretion by regulating MVBs morphogenesis. (A) HepAD38 cells were fixed and processed for detecting HBV (-) DNA, HBsAg and MVBs specific marker CD63. Specific enlargement of the area within the white-outlined box, colocalization of (-) DNA, HBsAg and CD63 were presented by white arrows. Scale bar, 4 μm. (B) Quantification of images in DMSO-treated cells and analyzing the relationship between the mean volume of CD63^+^ vesicles and (-) DNA puncta in each cell. (C, D) Mean volume and puncta of CD63^+^ vesicles in a single cell level were measured by Huygens. Analyzing (-) DNA and CD63^+^ positive (E), HBsAg and CD63^+^ positive (F) colocalization percent per cell within 3 μm. More than 15 cells per group was counted. ****P < 0.0001 (Mann-Whitney U-test).

A plethora of reports have shown that in the late steps of HBV replication, the egress of mature virions depends on intraluminal vesicles of maturing endosomes, i.e., multivesicular bodies (MVBs), and facilitated by various host factors. To further explore the microscale spatial relationship between MVBs and HBV virion secretion in vesicle fusion and budding and their dependence on MTs, we examined viral (-) DNA, HBsAg and CD63 (a marker for MVBs) in situ (Fig 7A). As shown in unperturbed condition (Fig 7A, upper panel), there existed a heterogeneity in the size of CD63^+^ vesicle. Furthermore, the number of viral (-) DNA puncta was negatively correlated with the mean volume of vesicles per cell, i.e., the smaller the vesicles, the more (-) DNA puncta per cell, and vice versa (Fig 7B). With MTIs treatment, the size distribution of CD63 ^+^ vesicles was altered (Fig 7A, middle and bottom). Quantitative analysis showed that Nocodazole and Vinblastine increased the number of vesicles (2.9 fold and 2.1 fold, respectively) and decreased their mean volumes per cell (46% and 58% decrease respectively) (Fig 7C and 7D). More importantly, the ratio of viral (-) DNA colocalized with CD63^+^ vesicles per cell increased by 2.3 fold and 2.2 fold after Nocodazole and Vinblastine treatment (Fig 7E). The colocalization ratio of HBsAg and CD63^+^ vesicles had the same trend in the presence of MTIs (Fig 7F).

Since MVBs emerge from fusion of early small endosomes into larger vesicles [29], the heterogeneity in the size of CD63^+^ vesicle and its negative correlation with the number of viral DNA in each cell suggested that virions were efficiently released once a large multivesicular body was formed. Consistently, the disruption of microtubule arrested early small vesicles transport, impeded their fusion into large MVBs and slowed down virion morphogenesis.

## Discussion

Recent improvements in FISH technology have enabled sensitive single-cell detection of viral nucleic acids in infected cells [30–32]. The sensitivity achieved by bDNA-FISH also lends itself well to diagnostic applications [33]. Importantly, the spatially resolved nature of FISH images provides key information on how viruses establish a successful infection by co-opting cellular machineries [30,32,34].

In this study, by combining bDNA-based FISH assay and specific nuclease treatment, we visualized major forms of viral nucleic acids, e.g., pgRNA, (-) DNA and intranuclear DNA with molecular specificity. We then used this assay for kinetic analysis. Unlike other viruses, the high level of inoculum in the early phase of the cell-based infection caused input background. Indeed, no significant difference in FISH signal was observed between live virus and UV-irradiated virus in the first three days. Nevertheless, we obtained signal from *de novo* synthesized RNA and DNA at later time points which exhibited considerable cell-to-cell variability. Interestingly, the statistics of RNA/DNA molecules per cell fitted well with geometric distribution which was quite different from other viruses such as HCV [30,35]. This could be due to reasons as follows: Firstly, HBV infection efficiency in the HepG2-NTCP system is low. Thus, the chance of receiving larger numbers of competent virions decreases logarithmically. Secondly, the HBV replication cycle required several slow steps such as transcription, encapsidation and DNA synthesis, which is in stark contrast to many RNA viruses, most of which do not have a nuclear phase. For instance, the incoming HCV RNA molecules can start translation and replication once they enter the cytoplasm which generates hundreds of progeny RNAs within 24 hours [30]. The preservation of geometric distribution even at later time points is probably the combined outcome of these factors.

We next used this assay to probe the nuclear reservoir of HBV and its colocalization with key epigenetic markers such as H3K27ac and Pol II using wide-field and super-resolution microscopy (STED). Colocalization of (+) DNA with H3K27ac and Pol II within nuclei were observed which supported the chromatinized nature and transcriptional activity of these molecules [36]. Further research is under way to interrogate the various epigenetic markers associated with cccDNA. In addition, we further confirmed the degradation of Smc5/6 complex in infected PHHs. By contrast, Smc5/6 in HepG2-NTCP cells was intact after HBV infection. This suggests that the Smc5/6-involved DNA damage repair mechanism may be significantly changed in hepatoma cell lines such as HepG2. Hence, enough attention should be paid to assess the suitability of cell models for studying HBx-mediated cellular responses.

We next determined colocalization of HBV pgRNA with actively translating HBV pgRNA. The resolution of our colocalization analysis was verified by CpAMs which caused core proteins to form abnormal capsids that were spatially segregated from pgRNA. Indeed, some have observed that capsid assembly inhibitors treatment results in the aberrant polymerization of core protein and the appearance of aggregated core polypeptides [24,25].

It is generally thought that viral RNA translation and nucleocapsid packaging are inherently competitive as indicated in research on different classes of viruses [30,37]. Furthermore, live-cell imaging of HIV-1 RNA showed that Gag proteins selectively package non-translating RNA into the assembly complex which provided time-resolved empirical evidence [37]. As to the HBV pregenome, it serves as the messenger for both core and Pol, which are synthesized at a relatively constant ratio (in the order of 100 to 1) [38,39]. As a result, the translation efficiency of pol is the rate limiting step for viral packaging and reverse transcription. Moreover, the C ORF initiation codon is located in the 5′ ε stem–loop structure which results in translation suppression when ε is properly folded [22,40]. Thus, it is generally thought that pgRNA translation and packaging are temporally sequential and spatially separate molecular events.

To experimentally test this theory, we quantitatively analyzed the number and spatial features of actively translating ribosome and encapsidated pgRNA. It was found that 60.5%-82.6% of the pgRNAs were free from capsid and ribosome. This suggested that a high percentage of transcribed pgRNAs are waiting for translation initiation. The high level of transcriptional activity in HepAD38 cells might be responsible for the higher proportion (82.6%) of free pgRNA in cytoplasm compared with that in HepG2-NTCP cells (60.5%). In addition, 64.1%-90.1% of the capsids were empty which significantly outnumbered immature nucleocapsid (9.1%-35.9%) which supported the mass production of empty capsids or empty virions as proposed [41].

In addition, there were 3.9 fold and 12.9 fold pgRNA/capsid colocalization puncta in relation to ribosome-occupied pgRNA in HepAD38 and HepG2-NTCP infection cell model, respectively. We inferred that it reflected the slow kinetics of nucleocapsid packaging, maturation as opposed to relatively quicker ribosome occupation and polypeptide synthesis. Indeed, it was estimated that around 6 amino acids are translated per second in eukaryotic cells [38,42]. Furthermore, the observed frequency of pgRNA colocalized with both capsid and ribosome was close to the estimate of random coincidence in HepAD38 cell model. But it was significantly higher than the estimate of random coincidence in HepG2-NTCP cell model. Indeed, pgRNA, capsid and translating ribosome were occasionally found to be clustered in proximity which could not be resolved by diffraction limited microscope. Nevertheless, it might suggest separate but subcellularly coupled process of pgRNA translation and encapsidation.

The microtubules play a key role in cargo and vesicles transport. Early and late endosomes traffic and fuse along endoplasmic reticulum and microtubules network with the recruitment of regulator proteins [43,44]. Many viruses (hepatitis C virus, influenza A virus and herpesvirus etc.) rely on MTs to transport viral genome or virus-loaded endosomes for their efficient replication and progeny virus formation [45–47]. A previous study suggested that MTs are crucial for nucleocapsid assembly and HBV replication [7]. However, our quantitative and spatial analysis indicated that although capsids were in proximity to MTs, their assembly was not affected by MTIs. Nevertheless, we observed accumulation and retention of nucleocapsids in the perinuclear region. Thus, microtubule integrity facilitates nucleocapsid transport but is dispensable for nucleocapsid assembly and viral DNA polymerization.

In addition to nucleocapsid transport, microtubule integrity was also found to be necessary for trafficking of virion containing vesicles. MTIs caused marked inhibition of mature virion release. FISH imaging revealed that mature virions were arrested in small CD63^+^ vesicles by MTIs treatment, which would normally fuse into larger MVBs. Thus, endosomal fusion into large intraluminal vesicles seems to be heavily dependent on microtubule-assisted transport. Indeed, plenty of evidence indicated that endosomes move along microtubules [48]. Moreover, their trafficking and maturation is coupled with microtubule-associated endoplasmic reticulum [43,49].

**Fig 8:**
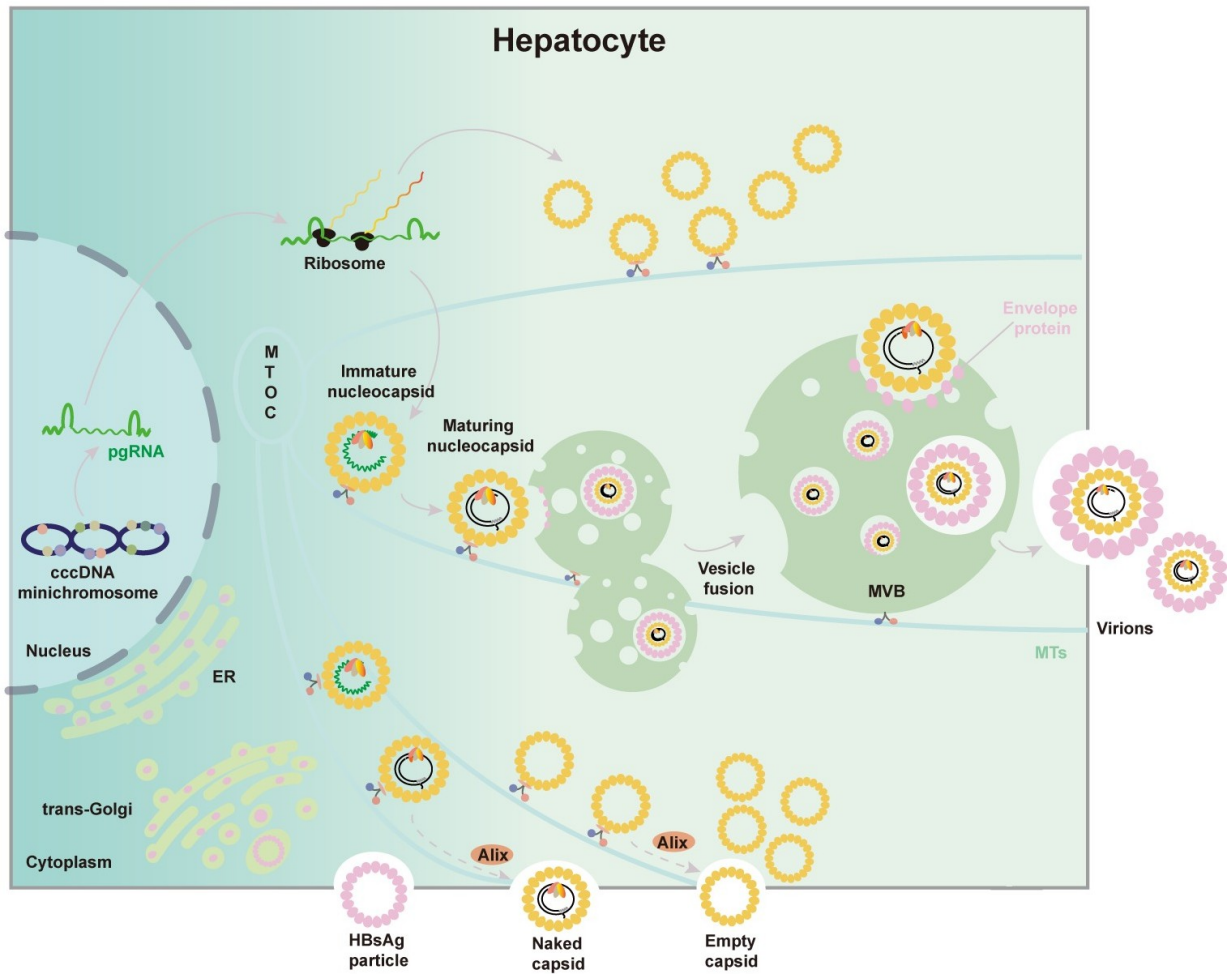
Schematic presentation of the molecular events from cccDNA transcription to virion morphogenesis based on the observations in this study.

In summary, a microscopic imaging platform was developed which enabled visualization of viral minichromosome and accompanied nuclear events critical for active transcription. Quantitative analysis of pgRNA suggested temporally sequential and spatially segregated translation and encapsidation. Due to the slow kinetics of viral DNA synthesis, core particle maturation is probably the rate limiting step for progeny virus production. In addition, we provided a novel view on how MTs orchestrate the integral process from HBV nucleocapsid assembly to virion morphogenesis and release. A proposed model of the HBV pgRNA translation, packaging and virion budding are illustrated according to our newly obtained data (Fig 8). The multiplexed nature of this methodology warrants further applications in various aspects of viral life cycle.

## Materials and methods

### Antibodies and compounds

Antibodies against HBcAg, preS1 and HBsAg were purchased from DAKO (B0586, CA), INNOVAX (M1055, China) and Fitzgerald (20-HR20, USA), respectively. Monoclonal antibodies against acetyl-Histone H3 (Lys27) (D5E4), RNA polymerase II (8WG16), Smc6 (M01), puromycin (4G11) and CD63 (H5C6) were purchased from Cell-Signaling Technology (8173, USA), Abcam (ab817, UK), Abgent (AT3956a, USA), Sigma-Aldrich (MABE342, MO) and BD Biosciences (556019, CA), respectively. Antibodies against Smc5, Lamin B1, α-tubulin and β-actin were purchased from Abcam (ab185373), Abcam (ab16048), Abcam (ab7291), and Sigma-Aldrich (SAB1305567), respectively. Alexa Fluor 488 labelled goat anti-mouse (ZF-0512) or anti-rabbit (ZF-0511) antibody were purchased from ZSGB-BIO (China). Cy3 labelled goat anti-mouse (115-165-146) or anti-rabbit (115-165-045) antibody were purchased from Jackson ImmunoResearch (USA). Polyclonal goat anti-mouse Ig-HRP (sc-2969) and anti-rabbit Ig-HRP (sc-2004) were purchased from Santa Cruz (USA).

Interferon-α was purchased from PBL Assay Science (11101–1, USA). Entecavir (HY-13623), Bay 41–4109 (HY-100029), GLS-4 (HY-108917), Nocodazole (HY-13520) and Vinblastine (HY-13780) were purchased from MedChemExpress (USA). Puromycin was purchased from Gene Operation (ISY 1130–100, USA).

### Cell culture, CCK8 assay

The HepG2-NTCP-A3 cells and HepAD38 cells were kindly provided by Prof. Stephan Urban (University of Heidelberg, Germany) and Prof. Jutao Guo (Blumberg Institute, USA) respectively. They were cultured as previously described [11,50]. Primary human hepatocytes (PHH, purchased from Celsis [Batch: AKB]) (Bioreclamation IVT) were cultured as previously described [51]. Mycoplasma contamination was routinely tested. For FISH experiment, cells were seeded on collagen-coated four-well Lab-Tek chamber slides (Thermo Fisher Scientific, USA). Cell viability was determined by the CCK8 assay kit (Dojindo, Japan) and performed as the manufacturer’s instructions.

### HBV infection

HBV was purified and concentrated 100-fold from the culture medium of stable HBV replication HepAD38 (DOX-) cells by ultrafiltration (Amicon Ultra, 100Kda, Millipore). Unless otherwise indicated, HepG2-NTCP-A3 cells were seeded on plates coated with collagen and infected with HBV at 1000 genome equivalents per cell in the presence of 2.5% DMSO and 4% PEG 8000 (Sigma-Aldrich) for 6–8 h. The inoculum was removed by extensive washing with PBS and infected cells were maintained in the medium containing 2.5% DMSO. The medium was changed every three days and samples were maintained until the preparation day at certain time points.

### Fluorescence in situ hybridization (FISH)

The FISH assay for HBV DNA and RNA was performed using the QuantiGene ViewRNA ISH cell assay kit (QVC0001, Thermo Fisher Scientific) according to the protocols with some modifications and three specific probe sets targeting HBV RNA and DNA were designed from Thermo Fisher Scientific (S1 Fig). Probe Set 1 (VF1-16020, Type 1; VF6-6000939, Type 6), which is complementary to the plus strand of the HBV sequence (nt 1932–2898, reference sequence: U95551.9), binds to pgRNA and plus-strand DNA. For the detection of pgRNA, we used DNase I (RNase-free) to eliminate plus-strand DNA interference. Probe Set 2 (VF6-6000421, Type 6), which is complementary to the minus strand of the HBV sequence (nt 2959–837, reference sequence: U95551.4), binds solely to minus-strand DNA. Probe Set 3 (VF6-6002152, Type 6), which is complementary to the gap region of the plus strand of HBV partial double-stranded genome (nt 500–1590, reference sequence: U95551.4). For the detection of intranuclear plus-stand DNA, we used RNase A/H to eliminate viral RNA.

Cells were fixed with 3.7% formaldehyde in DEPC-treated PBS for 10 min at room temperature (RT). Cells were then washed with PBS, permeabilized with 50% ethanol for 5 min and dehydrated in 100% ethanol at -20°C until use. Before hybridization, cells were rehydrated in 50% ethanol for 5 min, PBS for 10 min and 3.7% formaldehyde fixed the cells again. Cells were digested with RNaseA/H (detecting DNA) or DNase I (detecting RNA) at 37°C for 1 h. After washing with PBS and fixed (5min) once more with 3.7% formaldehyde fixed, specific probes were 100-fold diluted in Probe set diluent QF and added to the slide, which was covered by coverslips and sealed with rubber cement. Hybridization was performed with preheating at 75°C for 2 min and incubation at 40°C for 3 h in a humidified chamber. Washing and signal amplification were performed as described in the assay manual. Cells were counterstained with Hoechst33342 (Thermo Fisher Scientific) and finally mounted in fluorescence-antifade mounting medium (DAKO). The detailed FISH procedure can be obtained from the protocol database of ICE-HBV (https://ice-hbv.org/). For simultaneous FISH and immunofluorescence analyses, cells were blocked with 10% FBS and 0.1% Trixon-100 in PBS for 2 h at RT after label probe incubation and washing. Slides were then incubated with primary antibody followed by fluorophore labeled secondary antibody. They were finally counterstained and mounted as described above.

### Puromycylation of actively translating ribosomes

Cells seeded on collagen treated coverslips were incubated in media supplemented with 182 μM puromycin and 208 μM emetine (Sigma-Aldrich) for 5 minutes at 37°C. Cells were then incubated for 2 min on ice with permeabilization buffer (50 mM Tris-HCl [pH 7.5], 5 mM MgCl_2_, 25 mM KCl, 355 μM cycloheximide, EDTA-free protease inhibitors, 10 U/mL RNaseOut and 0.015% digitonin). After this extraction step, cells were washed with PBS, fixed in 3.7% formaldehyde and processed for FISH. Following pgRNA hybridization and blocking, cells were incubated with primary mouse anti-puromycin antibody and rabbit anti-HBcAg antibody, and then with goat anti-mouse Alexa Fluor 488-labeled secondary antibody and goat anti-rabbit Cy3. DNA was stained with Hoechst33342.

### Viral nucleic acid extraction, detection and quantification

HBV nucleocapsid DNA was extracted and detected by southern blot as described previously [13]. HBV RNA was extracted using TRIzol reagent and detected by northern blot as described [13]. Supernatant HBV DNA used for quantification was extracted using HBV DNA Quantitative Fluorescence Diagnostic Kit (Sansure Biotech, China) and determined by qPCR. Viral encapsidated-DNA and RNA were extracted and quantitatively detected according to the method described previously [52]. Total viral RNA from cells was extracted by TRIzol Reagent, followed with DNase I digestion to remove the remaining DNA and quantified with HBV RNA quantitative fluorescence assay kit (Sansure Biotech, China). For preC mRNA and pgRNA quantification, isolated HBV RNA was reverse transcribed into cDNA using PrimeScript RT reagent Kit with gDNA Eraser (Perfect Real Time) (Takara, RR047A). The sequences of preC mRNA specific forward primer, preC mRNA and pgRNA specific primer were 5’-GGAGGCTGTAGGCATAAATTGGTC-3’ (nt 1773–1797) and 5’-TGTGCCTTGGGTGGCTTT-3’ (nt 1875–1893), respectively. The reverse primer was the universal sequence: 5’-CGAGATTGAGATCTTCTGCGAC-3’ (nt 2434–2412). 1.3-fold full length HBV genome plasmid was used as the standard. The qPCR reaction mixture was from TB Green *Premix Ex Taq* (Tli RNaseH Plus), Bulk (Takara, RR420L).

### Viral antigen detection

Viral proteins were detected by immunoblotting and performed as previous described [52]. For supernatant HBsAg and HBeAg detection, the enzyme-linked immunosorbent assay kits (ELISA, Shanghai Kehua, China) were used.

### Nuclear extraction and immunoblotting

Cytoplasmic and nuclear fractions of HepG2-NTCP and PHH cells were isolated using a Nuclear and Cytoplasmic Protein Extraction Kit (Beyotime, P0028, Shanghai, China) and protein concentration was measured with BCA protein assay. In addition to Smc5 and Smc6, Lamin B1 and GAPDH were also detected as nuclear and cytoplasmic control respectively.

### Image acquisition and analysis

Most of images were acquired with an epifluorescence microscope (Olympus IX81) equipped with a 100× oil-immersion objective (NA 1.4, Olympus) and a 1.4 Megapixel sCMOS camera (Prime95B, Photometrics) with 95% max quantum efficiency. Images were taken in a series of Z steps at 0.3 μm intervals and across a range of 3 μm with a resolution of 1024 × 1024. A minority of the images were captured with a DeltaVision epifluorescence microscope (GE, USA) with a 100× oil-immersion objective (NA 1.4) and Photometrics CoolSNAP HQ2 CCD camera which were specifically indicated in figure legends. Images were deconvolved and analyzed using Huygens Essential software (Version 19.04, SVI, Netherlands) and processed using Fiji (ImageJ). STED super-resolution images were acquired with an 100x oil-immersion objective (Leica, NA 1.4) on Leica TCS SP8 STED inverted confocal microscope (Leica Microsystems, Germany) equipped with three excitation pulsed lasers (488, 561 and 633 nm) and STED depletion lasers (592, 660 and 775 nm) for detecting Alexa Fluor 488, Cy3 and Cy5, respectively. In brief, after correct collar position was set, images were first acquired in confocal mode with a pixel size of 100 nm, followed by STED imaging with a pixel size of 33 nm. Hybrid detector was used and 1–6 ns time gate were set to avoid reflection from the coverslip and undesired anti-Stokes background.

The DNA or RNA detection in FISH images was subjected to CellProfiler for cell segmentation, quantification of FISH dots per cell was performed using the FISH-quant package [53,54]. For quantitative colocalization analysis, the “Object Analyzer” module in Huygens Essential was used to calculate the distance between spots of one channel with their nearest neighboring spots of another channel. Colocalization were defined if the distance between the closest spots of two channels was within 500 nm. pgRNA under translation was defined as the colocalization of pgRNA with actively translating ribosomes. Encapsidated pgRNA (immature nucleocapsid) was defined as the colocalization of pgRNA with HBcAg. Minus strand (-) DNA and HBcAg colocalization was defined as maturing nucleocapsid and minus strand DNA colocalized with preS1/HBsAg was defined as nucleocapsid under envelopment.

### Statistical analysis

All data are expressed as means ± standard deviation (SD) or median. Statistical comparisons were made using a two-tailed Student’s *t*-test or Mann-Whitney U-test. P ≤ 0.05 was considered statistically significant. GraphPad Prism 7.0 and R packages (fitdistrplus and ggplot2) were used for distribution fitting and data visualization.

## Supporting information

S1 Fig Schematic illustration of probe set design for the detection of HBV pgRNA, (-) DNA and (+) DNA. Related to Figs 1–7.
(TIF)

S2 Fig Multiplexed quantification of (-) DNA and pgRNA in individual cells. **Related to Fig 2.** (A) The levels of preC mRNA, preC mRNA and pgRNA in HepAD38 (DOX-) and HBV-infected HepG2-NTCP cells were detected by RT-qPCR. (B) HepG2-NTCP cells were infected with HBV at MOI = 1000 and at the indicated times post-infection cells were fixed and processed for FISH detection. Scale bar, 4 μm. Intracellular nucleocapsid DNA, and total RNA were detected by Southern blot (C) and Northern blot (D), respectively.
(TIF)

S3 Fig Accumulation of HBV (-) DNA and pgRNA during infection. **Related to Fig 2.** The frequency distributions (Freq. dist) and cumulative distribution function (CDF) of FISH counts were derived from HBV (-) DNA (A) and pgRNA (B) at 6, 9, 12, 15 dpi. Black curves are the empirical result of the fit of the CDF of FISH counts, and red curves are the calculated geometric distribution. ^, median; *, average (mean).
(TIF)

S4 Fig HBV (-) DNA and pgRNA expression during antiviral drug treatment in the HepG2-NTCP infection system. **Related to Fig 2.** (A) Schematic of experimental procedure of drug treatment of HBV-infected HepG2-NTCP cells. Individual (-) DNA (B**)** and pgRNA (D) puncta per cell for each time point (6, 9, 12 dpi) were quantified during treatment with 1000 IU/mL Interferon-α (IFN-α) or 10 μM Entecavir (ETV). The percentage at the indicated time points for (-) DNA (C) and pgRNA (E) positive cells were quantified. **P < 0.01, ***P < 0.001. ns: no significance (Mann-Whitney U-test).
(TIF)

S5 Fig Quantification of the colocalization between (+) DNA, H3K27ac and Pol II of Fig 3C. Inter-relationship among (+) DNA, H3K27ac and Pol II in HepG2-NTCP infection system illustrated by Venn diagram.
(TIF)

S6 Fig HBV DNA inversely correlates with Smc5/6 in HBV- infected PHH. (A) Isolated Smc5 and Smc6 proteins from whole cell lysate, cytoplasmic and nuclear fractions of HepG2-NTCP and PHH cells were detected by immunoblotting with the same protein loading amount. (B) HepG2-NTCP and HBV-infected HepG2-NTCP cells were fixed and processed for (-) DNA detection followed by immunofluorescence staining for Smc5 with Alexa fluor 488 labelled goat anti-mouse secondary antibody (upper panel). PHH and HBV-infected PHH were detected for (-) DNA and Smc6 (bottom panel). Scale bar, 4 μm. Smc6-positive and (-) DNA-negative cells were indicated by solid white arrows and autofluorescence were indicated by thin white arrows (Inset of the bottom panel). (C) (-) DNA puncta were quantified by FISH-quant. More than 30 cells per group were counted.
(TIF)

S7 Fig The specificity of puromycin labeling in HepG2-NTCP and HepAD38 cells. **Related to Fig 4 & 5**. Cells were untreated (A, C) or pretreated with Puromycin followed by cycloheximide (B, D) and processed for immunofluorescence using the primary anti-puromycin monoclonal antibody and Alexa Fluor 488 labelled goat anti-mouse secondary antibody and anti-core antibody with Cy3 labelled goat anti-rabbit antibody. Scale bar, 4 μm.
(TIF)

S8 Fig Replication of HBV after capsid assembly inhibitors treatment in HepAD38 (DOX-) cell. **Related to Fig 4.** HepAD38 (DOX-) cells were treated with Bay 41–4109 or GLS4. After 3 days, extracellular HBV-DNA (A), intracellular HBV encapsidated pgRNA (B), and intracellular HBV nucleocapsid DNA (C) were quantified by qPCR. (D) Intracellular nucleocapsid DNA was detected by Southern blot.
(TIF)

S9 Fig Inter-relationship among pgRNA, capsid and ribosome in HepAD38 cells and HepG2-NTCP infection system illustrated by Venn diagram. Related to Fig 5.
(TIF)

S10 Fig The effects of MTIs on cell viability, tubulin expression and intracellular pgRNA and (-) DNA. **Related to Fig 6.** (A) Cell viability under Nocodazole and Vinblastine treatment after 24 h was determined by CCK8 assay. Their effects on cellular α-tubulin expression (B), HBV pgRNA (C) and (-) DNA (D) were analyzed. For (C-D), a total of 150 cells per group was counted and the puncta per cell were shown as box plots. ***P < 0.001, ****P < 0.0001. ns: no significance. (A): Student’s *t*-test, the data are representative of three independent replicates; (C, D): Mann-Whitney U-test.
(TIF)

S11 Fig The effects of MTIs on virion morphogenesis. **Related to Fig 7.** HepAD38 cells were treated with Nocodazole and Vinblastine for 24 h, intracellular LHBsAg (A) (*, non-specific band), HBsAg and HBeAg in supernatant (B) and viral DNA in supernatant (C) were quantified by immunoblotting, ELISA and real-time PCR respectively. (D) Intracellular distribution of HBV (-) DNA and preS1 were visualized by FISH and immunofluorescence. White arrows indicate (-) DNA puncta colocalizing with preS1. Scale bar, 4 μm. (E) The number of mature virions (DNA, preS1 double positive) per cell were shown as dot plot. More than 15 cells per group was counted. **P < 0.01, ***P < 0.001. ns: no significance (B, C: Student’s *t*-test, the data are representative of three independent replicates; E: Mann-Whitney U-test).
(TIF)
