# Supplementary figures and images for: Probing the spatiotemporal patterns of HBV multiplication reveals novel features of its subcellular processes
Source: PLoS Pathog. 2021 Aug 9;17(8):e1009838. doi: 10.1371/journal.ppat.1009838 (PMC8376071; doi:10.1371/journal.ppat.1009838)

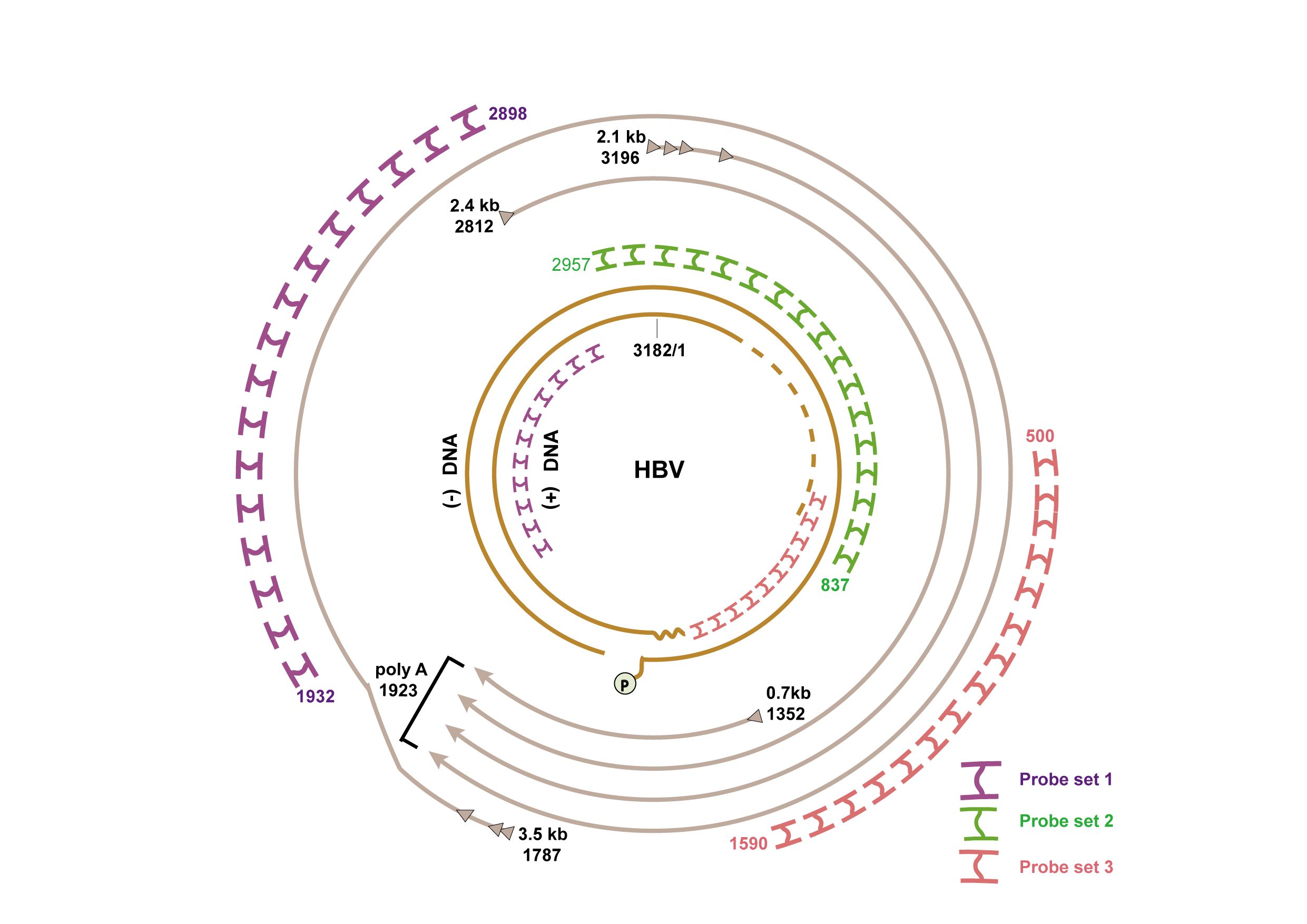

Supplement: S1 Fig — Related to Figs 1–7. (TIF) [file ppat.1009838.s001.tif]

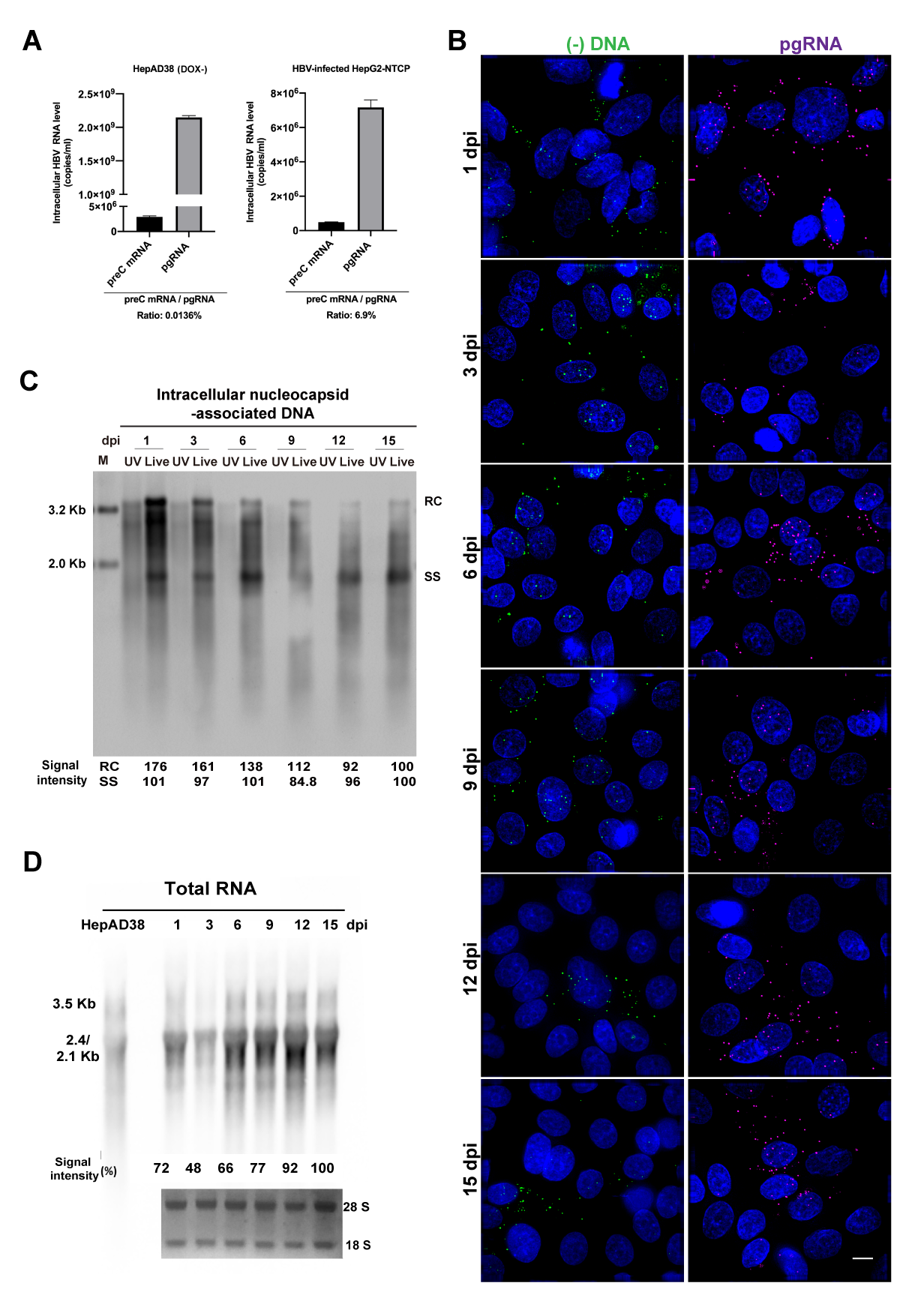

Supplement: S2 Fig — Related to Fig 2. (A) The levels of preC mRNA, preC mRNA and pgRNA in HepAD38 (DOX-) and HBV-infected HepG2-NTCP cells were detected by RT-qPCR. (B) HepG2-NTCP cells were infected with HBV at MOI = 1000 and at the indicated times post-infection cells were fixed and processed for FISH detection. Scale bar, 4 μm. Intracellular nucleocapsid DNA, and total RNA were detected by Southern blot (C) and Northern blot (D), respectively. (TIF) [file ppat.1009838.s002.tif]

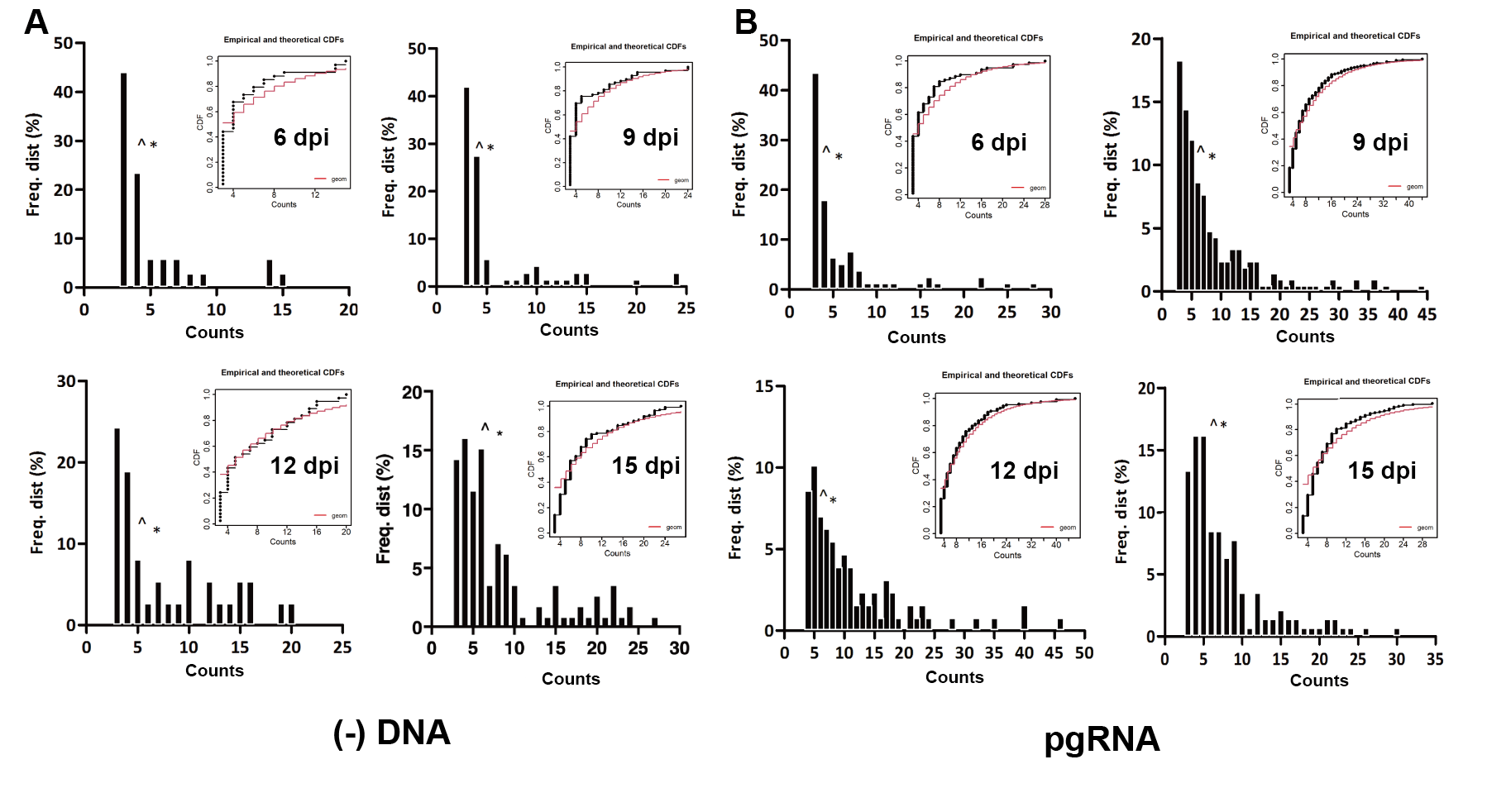

Supplement: S3 Fig — Related to Fig 2. The frequency distributions (Freq. dist) and cumulative distribution function (CDF) of FISH counts were derived from HBV (-) DNA (A) and pgRNA (B) at 6, 9, 12, 15 dpi. Black curves are the empirical result of the fit of the CDF of FISH counts, and red curves are the calculated geometric distribution. ^, median; *, average (mean). (TIF) [file ppat.1009838.s003.tif]

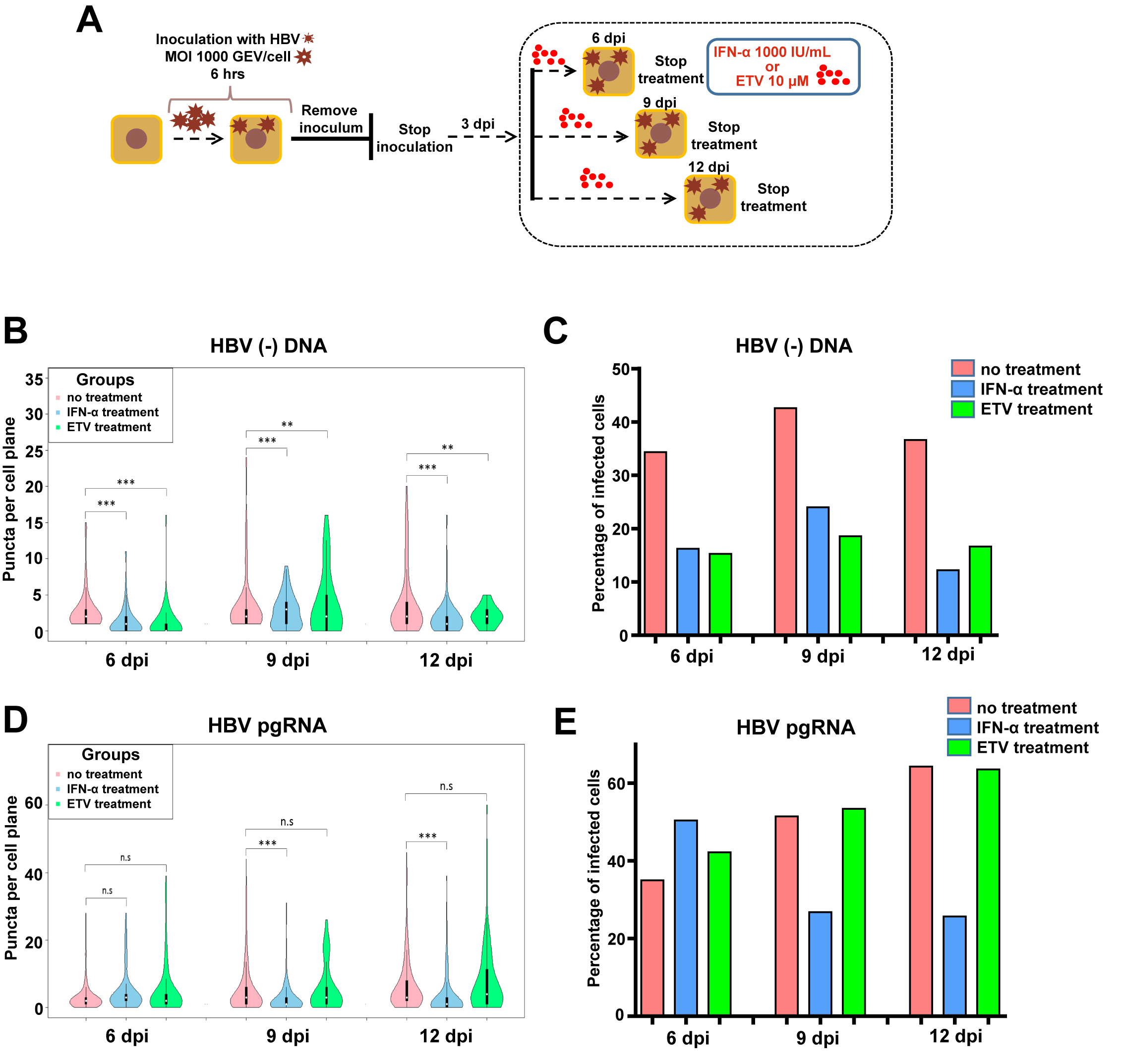

Supplement: S4 Fig — Related to Fig 2. (A) Schematic of experimental procedure of drug treatment of HBV-infected HepG2-NTCP cells. Individual (-) DNA (B) and pgRNA (D) puncta per cell for each time point (6, 9, 12 dpi) were quantified during treatment with 1000 IU/mL Interferon-α (IFN-α) or 10 μM Entecavir (ETV). The percentage at the indicated time points for (-) DNA (C) and pgRNA (E) positive cells were quantified. **P < 0.01, ***P < 0.001. ns: no significance (Mann-Whitney U-test). (TIF) [file ppat.1009838.s004.tif]

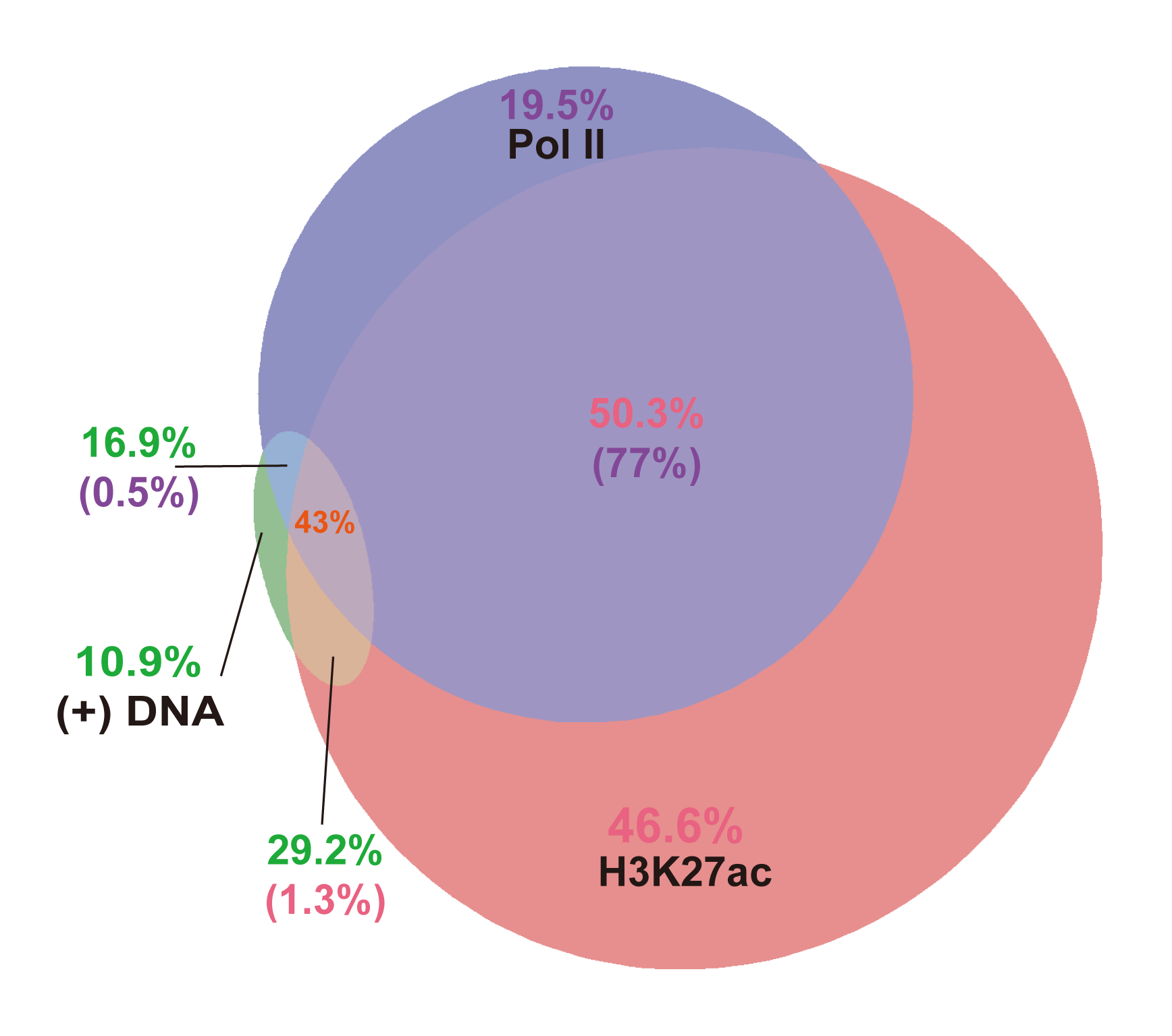

Supplement: S5 Fig — Inter-relationship among (+) DNA, H3K27ac and Pol II in HepG2-NTCP infection system illustrated by Venn diagram. (TIF) [file ppat.1009838.s005.tif]

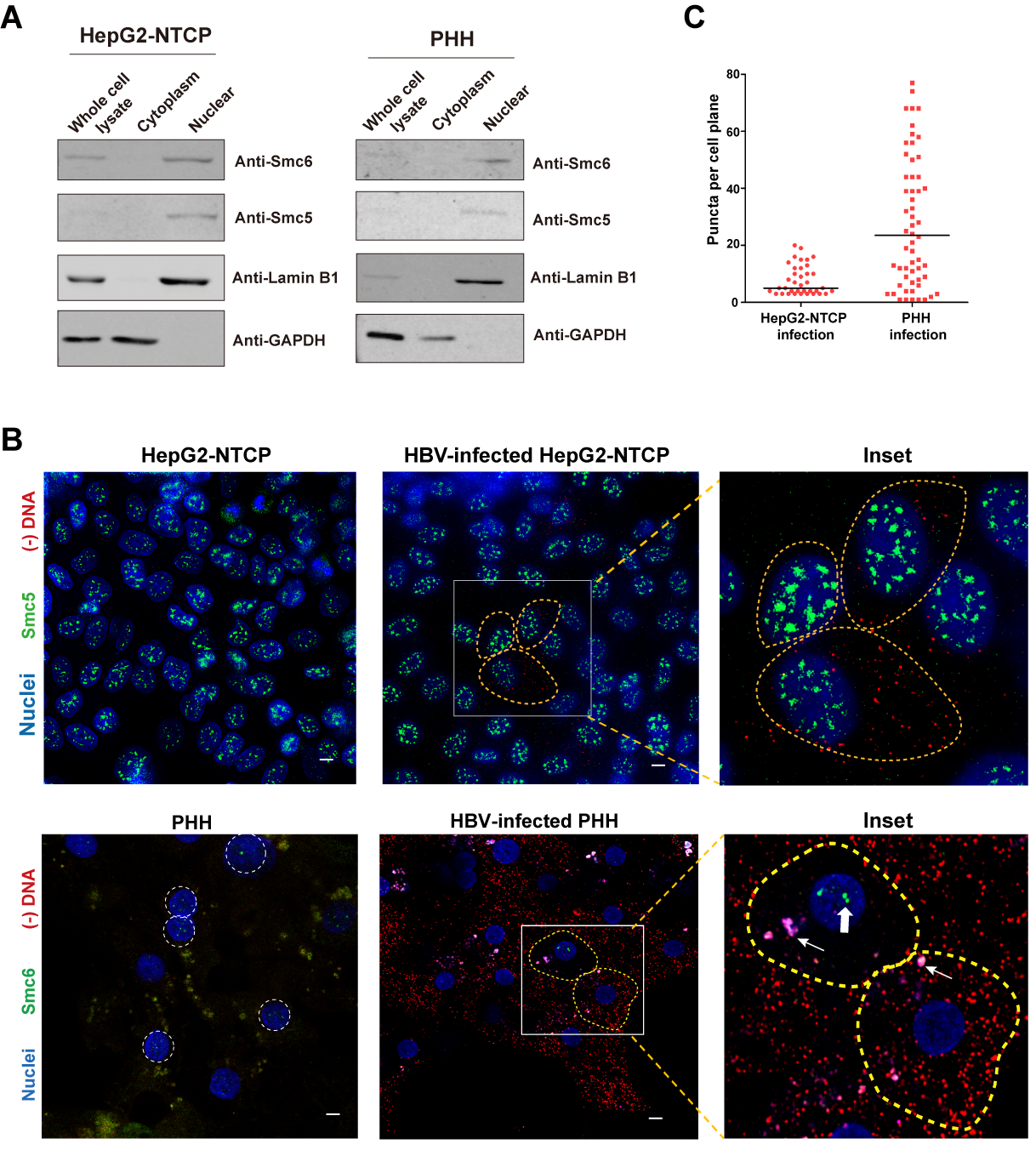

Supplement: S6 Fig — (A) Isolated Smc5 and Smc6 proteins from whole cell lysate, cytoplasmic and nuclear fractions of HepG2-NTCP and PHH cells were detected by immunoblotting with the same protein loading amount. (B) HepG2-NTCP and HBV-infected HepG2-NTCP cells were fixed and processed for (-) DNA detection followed by immunofluorescence staining for Smc5 with Alexa fluor 488 labelled goat anti-mouse secondary antibody (upper panel). PHH and HBV-infected PHH were detected for (-) DNA and Smc6 (bottom panel). Scale bar, 4 μm. Smc6-positive and (-) DNA-negative cells were indicated by solid white arrows and autofluorescence were indicated by thin white arrows (Inset of the bottom panel). (C) (-) DNA puncta were quantified by FISH-quant. More than 30 cells per group were counted. (TIF) [file ppat.1009838.s006.tif]

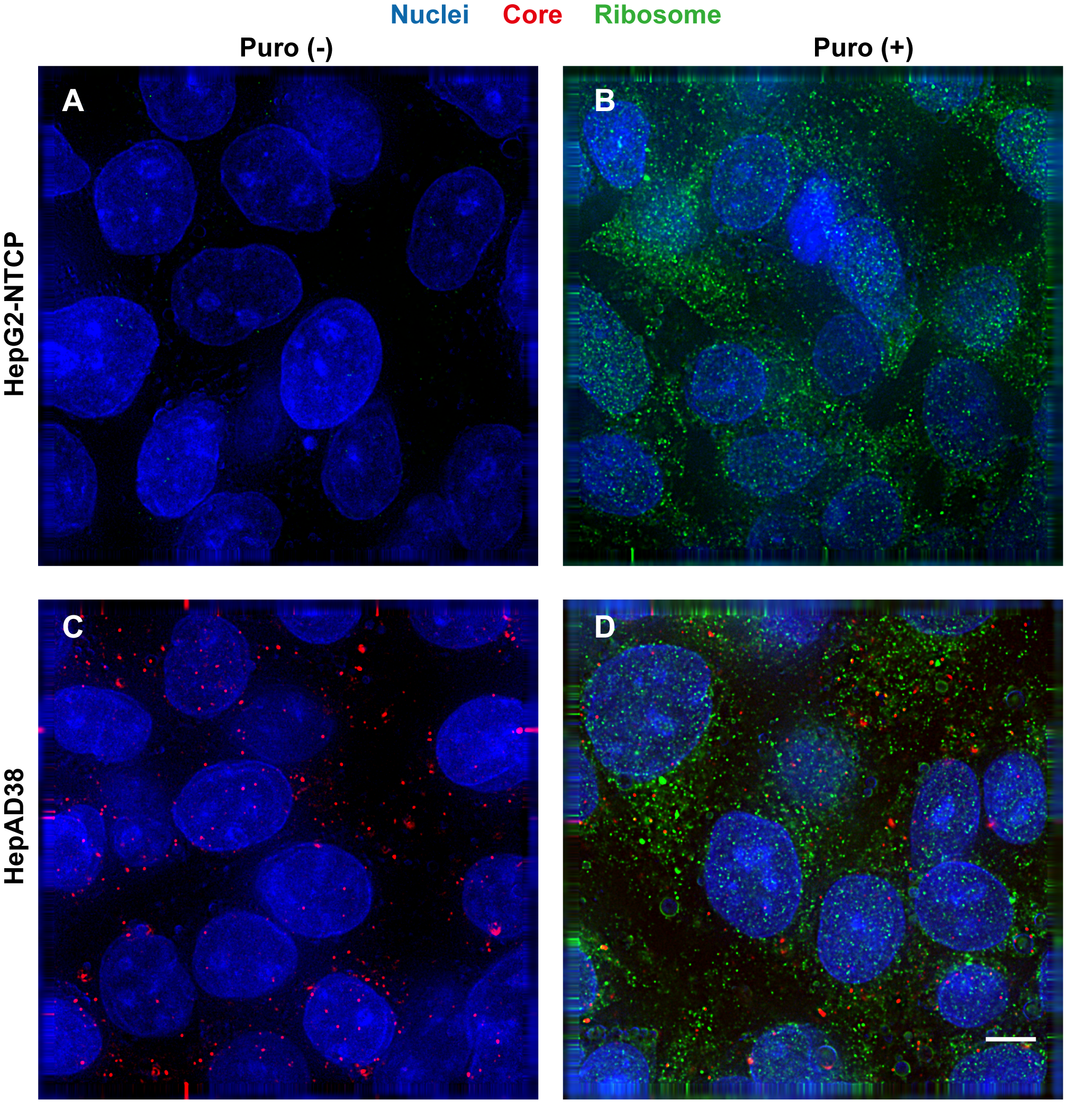

Supplement: S7 Fig — Related to Fig 4 & 5. Cells were untreated (A, C) or pretreated with Puromycin followed by cycloheximide (B, D) and processed for immunofluorescence using the primary anti-puromycin monoclonal antibody and Alexa Fluor 488 labelled goat anti-mouse secondary antibody and anti-core antibody with Cy3 labelled goat anti-rabbit antibody. Scale bar, 4 μm. (TIF) [file ppat.1009838.s007.tif]

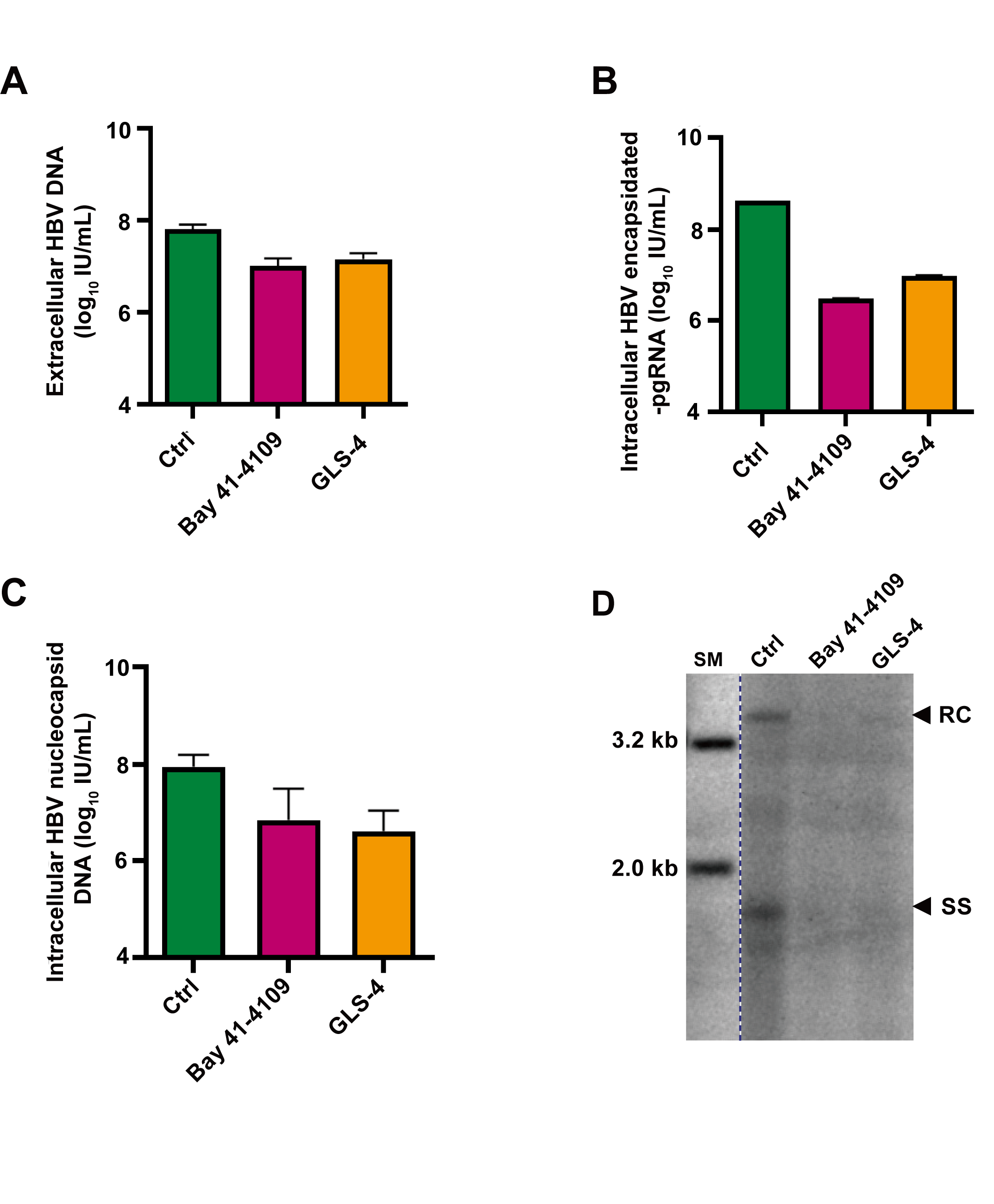

Supplement: S8 Fig — Related to Fig 4. HepAD38 (DOX-) cells were treated with Bay 41–4109 or GLS4. After 3 days, extracellular HBV-DNA (A), intracellular HBV encapsidated pgRNA (B), and intracellular HBV nucleocapsid DNA (C) were quantified by qPCR. (D) Intracellular nucleocapsid DNA was detected by Southern blot. (TIF) [file ppat.1009838.s008.tif]

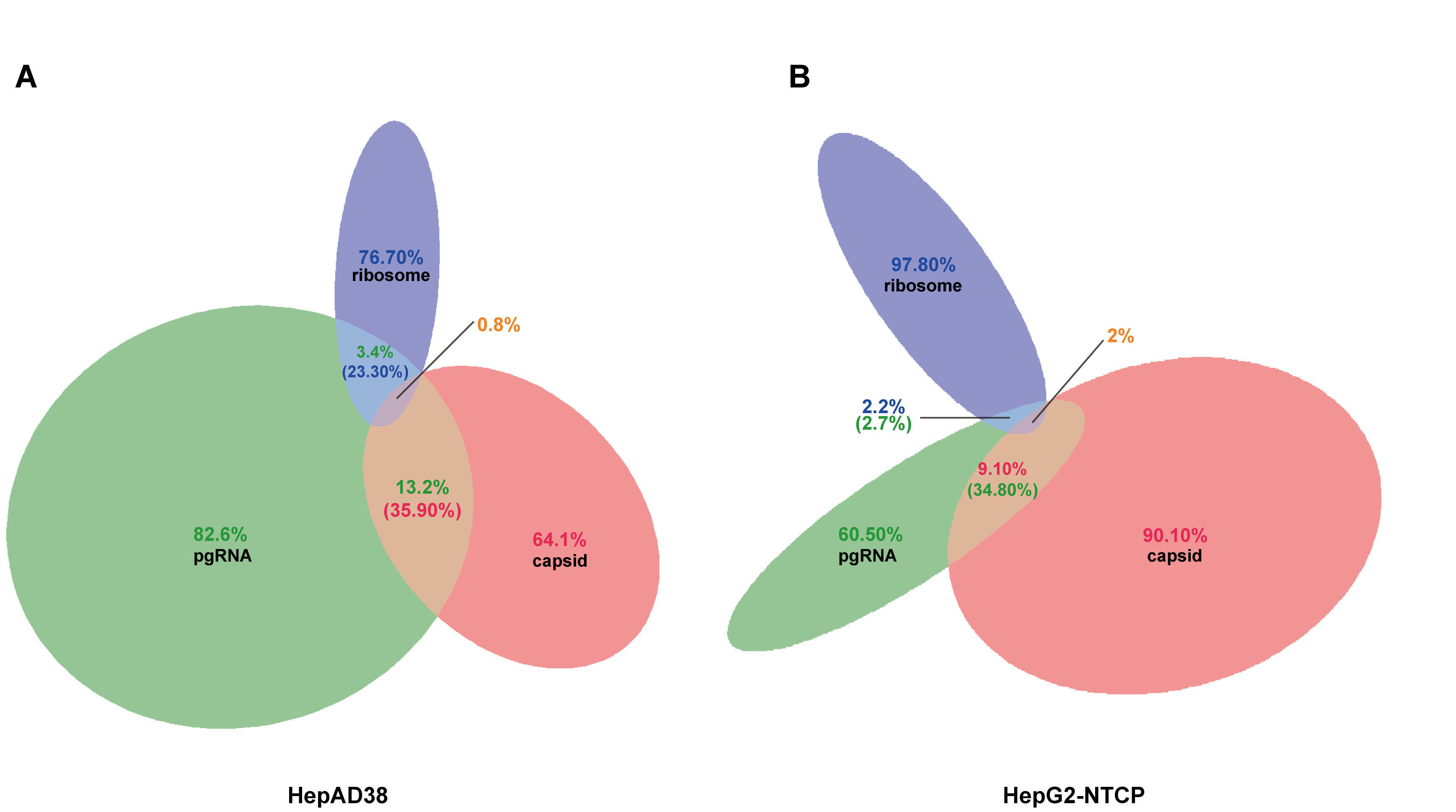

Supplement: S9 Fig — Related to Fig 5. (TIF) [file ppat.1009838.s009.tif]

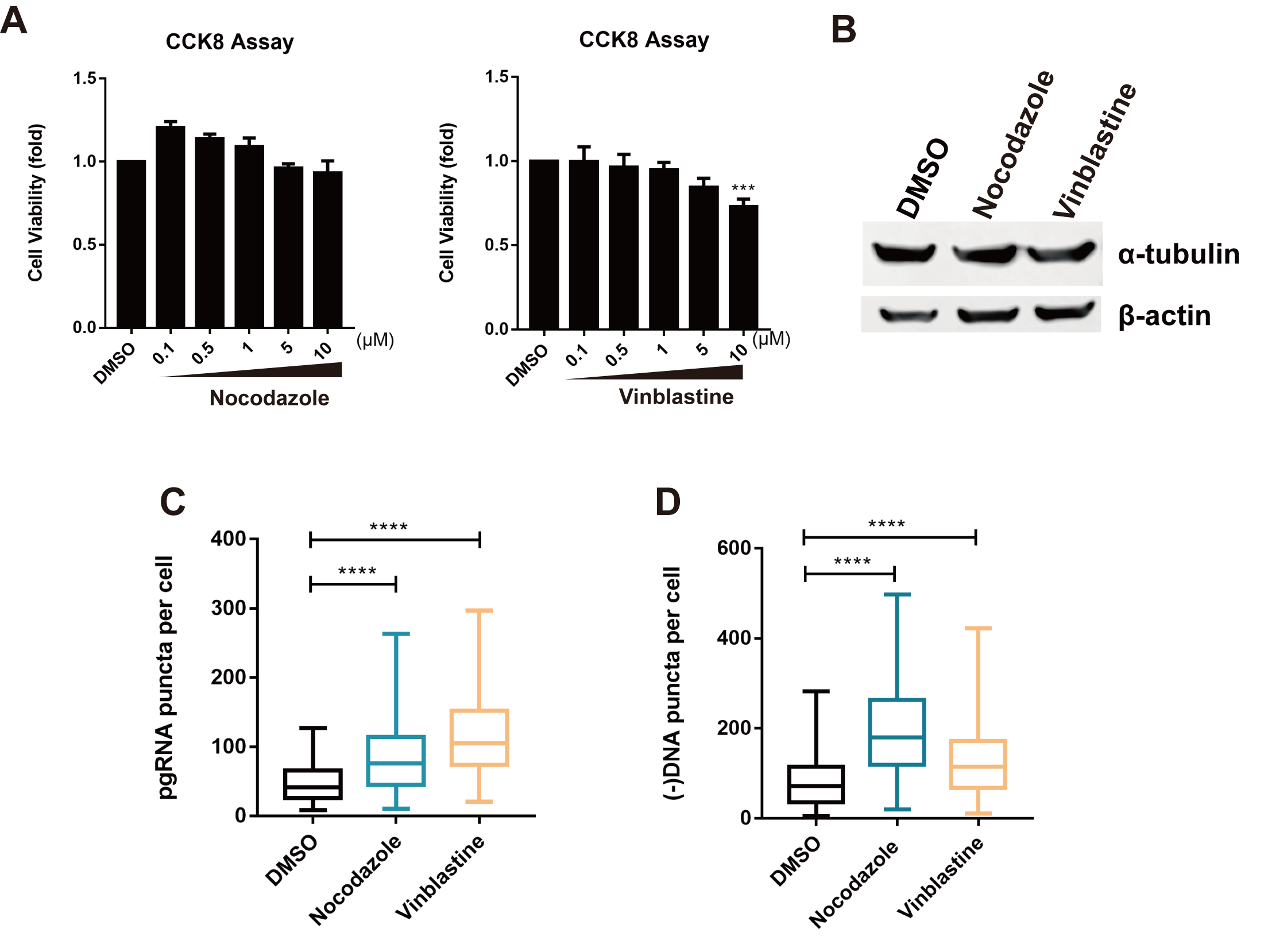

Supplement: S10 Fig — Related to Fig 6. (A) Cell viability under Nocodazole and Vinblastine treatment after 24 h was determined by CCK8 assay. Their effects on cellular α-tubulin expression (B), HBV pgRNA (C) and (-) DNA (D) were analyzed. For (C-D), a total of 150 cells per group was counted and the puncta per cell were shown as box plots. ***P < 0.001, ****P < 0.0001. ns: no significance. (A): Student’s t-test, the data are representative of three independent replicates; (C, D): Mann-Whitney U-test. (TIF) [file ppat.1009838.s010.tif]

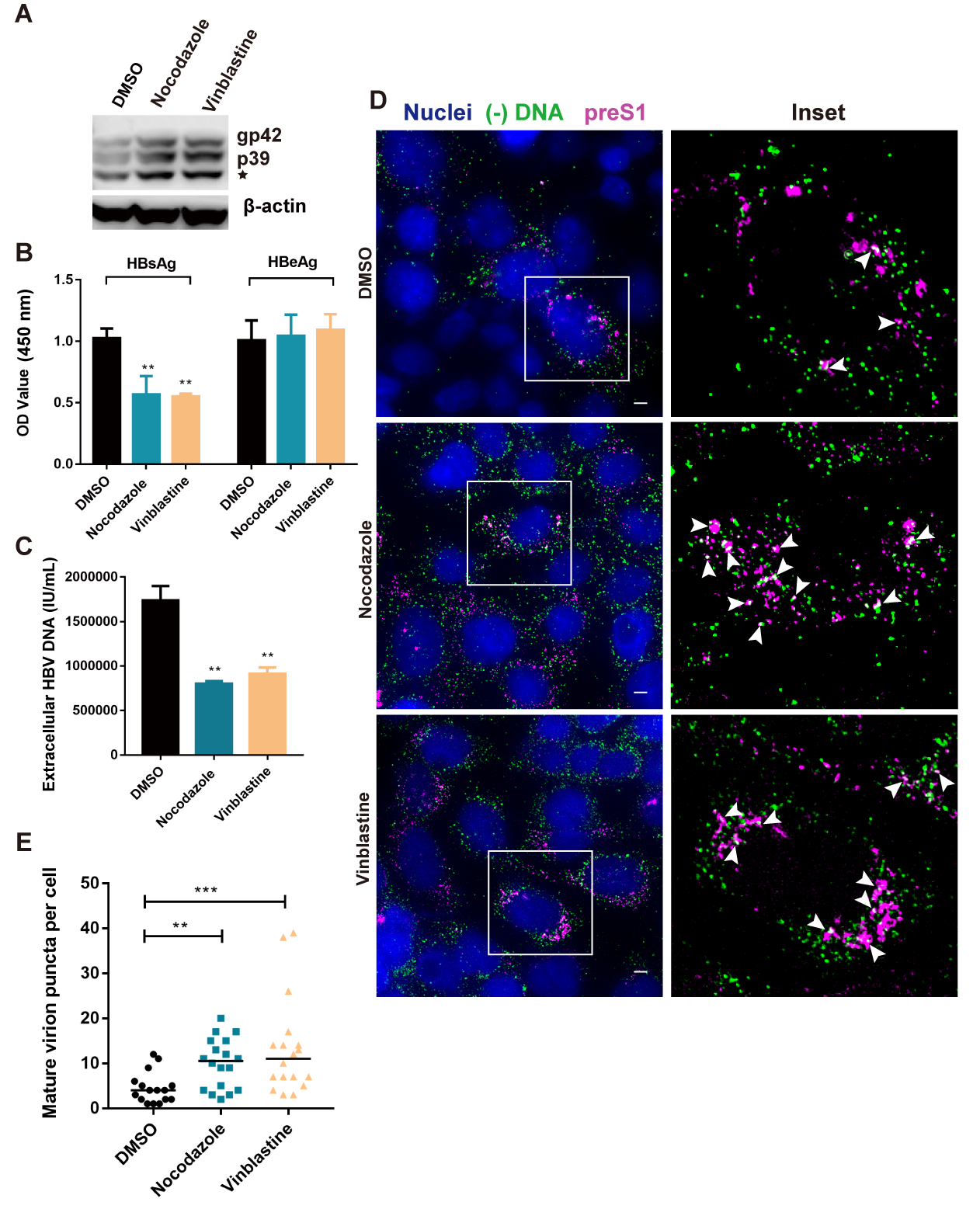

Supplement: S11 Fig — Related to Fig 7. HepAD38 cells were treated with Nocodazole and Vinblastine for 24 h, intracellular LHBsAg (A) (*, non-specific band), HBsAg and HBeAg in supernatant (B) and viral DNA in supernatant (C) were quantified by immunoblotting, ELISA and real-time PCR respectively. (D) Intracellular distribution of HBV (-) DNA and preS1 were visualized by FISH and immunofluorescence. White arrows indicate (-) DNA puncta colocalizing with preS1. Scale bar, 4 μm. (E) The number of mature virions (DNA, preS1 double positive) per cell were shown as dot plot. More than 15 cells per group was counted. **P < 0.01, ***P < 0.001. ns: no significance (B, C: Student’s t-test, the data are representative of three independent replicates; E: Mann-Whitney U-test). (TIF) [file ppat.1009838.s011.tif]
